# Discovery of Intermediary Genes between Pathways Using Sparse Regression

**DOI:** 10.1371/journal.pone.0137222

**Published:** 2015-09-08

**Authors:** Kuo-ching Liang, Ashwini Patil, Kenta Nakai

**Affiliations:** Institute of Medical Science, The University of Tokyo, 4-6-1 Shirokanedai, Minato-ku, Tokyo 108-8639, Japan; Louisiana State University Agricultural Center, UNITED STATES

## Abstract

The use of pathways and gene interaction networks for the analysis of differential expression experiments has allowed us to highlight the differences in gene expression profiles between samples in a systems biology perspective. The usefulness and accuracy of pathway analysis critically depend on our understanding of how genes interact with one another. That knowledge is continuously improving due to advances in next generation sequencing technologies and in computational methods. While most approaches treat each of them as independent entities, pathways actually coordinate to perform essential functions in a cell. In this work, we propose a methodology based on a sparse regression approach to find genes that act as intermediary to and interact with two pathways. We model each gene in a pathway using a set of predictor genes, and a connection is formed between the pathway gene and a predictor gene if the sparse regression coefficient corresponding to the predictor gene is non-zero. A predictor gene is a shared neighbor gene of two pathways if it is connected to at least one gene in each pathway. We compare the sparse regression approach to Weighted Correlation Network Analysis and a correlation distance based approach using time-course RNA-Seq data for dendritic cell from wild type, MyD88-knockout, and TRIF-knockout mice, and a set of RNA-Seq data from 60 Caucasian individuals. For the sparse regression approach, we found overrepresented functions for shared neighbor genes between TLR-signaling pathway and antigen processing and presentation, apoptosis, and Jak-Stat pathways that are supported by prior research, and compares favorably to Weighted Correlation Network Analysis in cases where the gene association signals are weak.

## Introduction

Genes in eukaryotic genomes rarely work alone, rather, they cooperate and interact with other genes to form networks or pathways. Gene products can act as activators or repressors to other genes, or bind with each other to form more complicated structures. Many types of pathways or interaction databases have been made available, such as databases for metabolic pathways [1, 2], signal transduction pathways [3, 4], and protein-protein interaction networks [5, 6].

The use of pathways in gene, protein, and genome structural variation analyses has become increasingly important as our understanding of the networks and pathways improved with recent advances in high-throughput technology. It has allowed researchers to make sense of observations about the expressions of genes or proteins not only as singular events, but also in a broader context of what is occurring in their interaction neighborhoods. Our current approaches for analyzing gene expression profiles using pathways often rely on finding pathways with an overrepresentation of differentially expressed genes [7]. In these types of analyses, a pathway is seen as a collection of genes independent from other genes and pathways. However, pathways often work together as a cascade of pathways for the transduction of biological signals and for other cellular functions. Therefore, while much progress has been made in the understanding of individual pathways, pathway-based analyses are often affected by the interaction or crosstalk that exist between different pathways [8]. Given that current knowledge of gene interactions is still incomplete, many interactions may still exist between upstream and downstream pathways. Finding these genes to fill in the missing pieces of the puzzle is crucial to the full understanding of the interactive pathways in our genome.

Correlation of gene expression is a common approach for finding novel gene interactions [9–11], but it can be sensitive to sample size [12]. In this work, we propose a sparse regression-based methodology, aimed at discovering intermediary genes between two pathways. For the analysis of pathways 1 and 2, the proposed method divides genes into three gene sets: genes in pathway 1, genes in pathway 2, and the remaining genes. It looks for genes in the remaining set that are associated with genes in both pathways, i.e., shared neighbor genes of the two pathways. More specifically, we use sparse regression with the remaining genes as predictors to model genes in pathways 1 and 2. Predictor genes having non-negative coefficients are considered as having interactions with the modeled pathway genes.

For a knockout experiment, we can further compare the shared neighbor genes found in the wild type and knockout samples, in order to find shared neighbor genes that are uniquely affected by the knockouts. Comparison of changes in shared neighbor genes can lead to discovery of genes that are essential for the communication between the two pathways.

## Materials and Methods

In this work, we will formulate the proposed methodology for RNA-Seq gene expression profile experiments. The method is applied to RNA-Seq data to discover shared neighbor genes, but can also used with any technology that measures the gene expression profile of a sample.

### Dataset

We use two RNA-Seq datasets to evaluate the proposed method for the prediction of shared neighbor genes between two pathways. The first dataset is a time-series gene-knockout experiment of mouse dendritic cells, which was previously made public in [13]. The second set consists of RNA-Seq data of 60 Caucasian individuals [14] obtained from [15].

#### Mouse Dendritic Cell Knock-out Time-course Data

In adaptive immune response, dendritic cells act as intermediary between antigens and mammalian immune mechanism by processing and presenting antigens to lymphocytes. One of the most important pathways involved in the activation of innate immune response is the Toll-like receptor 4 (TLR4) signaling pathway. TLR-4 signaling pathway is activated when lipopolysaccharide (LPS) found on the surface of Gram-negative bacteria is bound to the extracellular domain of TLR4, which eventually leads to the activation of proinflammatory cytokines and type-1 interferons [16]. After LPS binding, TLR4 signaling branches into two pathways, independently utilizing the adaptor proteins MyD88 and TRIF [17]. MyD88-dependent pathway is utilized for the rapid activation of IRAK1, IRAK4, and TAK1, which are important for the activation of MAPK and NF-*κ*B genes, whereas the TRIF-dependent pathway is essential for the production of interferon-*β* and late-phase activation of NF-*κ*B [18]. Understanding how the two independent pathways interact with downstream activities, and finding genes that are involved in signal transduction between the upstream and downstream pathways, are important steps for further understanding of mammalian adaptive immune response. In this work we use a dataset that consists of wild type, MyD88 KO, and TRIF KO mouse dendritic cell samples. Each sample was extracted from bone-marrow cells under the presence of GM-CSF. All three types of cells were then stimulated with LPS to elicit immune response. Samples from the stimulated cells were collected at 0hr, 0.5hr, 1hr, 2hrs, 3hrs, 4hrs, 6hrs, 8hrs, 16hrs, and 24hrs after stimulation, and RNA-Seq was performed on each sample. The time-series RNA-Seq data is currently available in Sequence Read Archive with accession number DRA001131 [13].

Prior to analysis by the proposed method, the mouse dendritic cell time-course RNA-Seq dataset was checked for read quality using FastQC [19]. The resulting reads for each of the three cell types were mapped to *M. musculus* mm10 genome RefSeq gene annotations using Bowtie1 [20] and Tophat2 [21]. Indices and annotations for Bowtie1 and Tophat2 were downloaded from the respective programs’ websites. Per-base read quality scores and mapping rates for each sample are shown in S1 Fig. Reads that were successfully mapped by Tophat2 to the mouse transcriptome were then used to estimate the gene expressions in each time sample using Cufflinks [22]. Gene expression across different time samples in the same cell type were normalized as FPKM (fragment per kilobase of exon per million fragments mapped) and as a time series using Cuffdiff with option -T [22].

Before analyzing the processed RNA-Seq data, we first filtered out genes that have no expression or show limited changes in expression throughout the time series in all three cell types. We kept for subsequent analysis only those genes that in at least one of the cell types have a greater than 2-fold change between the maximum and minimum expressions, and have a maximum expression of greater than 5 fpkm. The remaining 5,676 genes were then z-normalized to mean of zero and variance of one.

#### Caucasian RNA-Seq Data

To test the robustness of the proposed method under different sample sizes, we have included RNA-Seq sequenced from mRNA obtained from the lymphoblastoid cell lines (LCL) of 60 Caucasian extended HapMap individuals [14]. The raw read count of each gene has been compiled and made available on the ReCount website (http://bowtie-bio.sourceforge.net/recount/) [15]. The raw read counts in a sample were normalized by dividing by the 75th percentile of the non-zero read counts of that sample [15, 23]. The total number of genes was further filtered down to 3,599 genes by requiring that the gene have at least one read in each of the 60 samples. Since these samples were not stimulated with LPS, they further test the sensitivity of the proposed method in datasets with weak signals.

### Gene expression profile similarity

To determine which genes are important to the transduction of signals between two distinct pathways, we make the assumption that genes with similar expression profiles are more likely to be interacting with each other. Given gene expression data from RNA-Seq experiments, one way of determining the similarity between two genes is to compute some measure of distance between their expression profiles. One of the most commonly used distance measures is the correlation distance, based on either the Spearman or the Pearson correlation coefficients. Non-correlation-based distance measures such Euclidean, City-block, and Maximum distance, which are all special cases of Minkowski distances [24], have also been used as distance measures in clustering algorithms for gene expressions [25]. For time-series gene expression data, Mahalanobis distance has been used as a distance measure to detect differential expression [26].

There is a vast amount of literature dealing with using distance measures to determine the similarity of two genes based on their gene expression. However, as with all distance-based approach, performance of each distance metric can be affected by the distribution and noise of the data. In particular, Euclidean distance does not consider correlation between data, whereas sample correlation coefficient is sensitive to outliers and sample size [27]. In this work, instead of using a distance measure and choosing a threshold for calling gene interaction, we propose the use of a sparse regression approach called elastic-net to predict gene interaction.

### Sparse regression

Current advances in sequencing technology have led to a tremendous growth in biomedical data, where sample dimensions, such as the number of genes or SNPs, have been growing at a much faster rate than that of number of samples. This phenomenon has emphasized the need for dimensionality reduction techniques in order to enhance the interpretability of statistical models used to analyze these data [28, 29]. One such example is lasso, a sparse regression model based on the ℓ_1_ norm, that has been applied to many problems in bioinformatics and computational biology [30].

One disadvantage of lasso regression is that it selects at most *T* predictors with non-zero coefficients, where *T* is the number of samples [31]. In the case where there are *P* predictors and *P* ≫ *T*, lasso regression may not be able to select enough predictors to model the dependent variable. Also, lasso may randomly select one variable from a group of variables with high pairwise correlations. In this work, we will use a sparse regression that overcomes these limitations by linearly combining the ℓ_1_ and ℓ_2_ penalties as regularization terms [31]. This sparse regression, or elastic-net, has the following optimization function:

$$\begin{array}{c} {\hat{\omega }}^{EN}=\underset{\omega }{\text{arg}\;\text{min}}({∥y-X\omega ∥}^{2}+(1-\lambda ){∥\omega ∥}^{2}+\lambda {∥\omega ∥}_{1}), \end{array}$$ (1)

where ***y*** is the dependent variable, ***X*** is the independent variable or predictor, ***ω*** is the coefficient of the predictor, and *λ* is a parameter that determines the sparsity of the model fitting.

In our work, genes *a* and *b* are defined as neighbor genes if they have “similar” expression profile. If gene *a* and gene *b* are neighbors and gene *a* belongs to pathway 1, then gene *b* is also considered as a neighbor of pathway 1. Let us first define **Ω** as the set of all genes, ***χ***_1_ as the set of genes in pathway 1, and ***χ***_2_ as the set of genes in pathway 2, and there is no overlap between ***χ***_1_ and ***χ***_2_, i.e. ***χ***_1_ ∩ ***χ***_2_ = ∅. We can further define **Π** as the set of all genes not in pathway 1 and pathway 2, or **Π** = (**Ω**\***χ***_1_) ∩ (**Ω**\***χ***_2_), where \ denotes set difference. Then, our goal is to find the set of genes **Γ**_1_ ∈ **Π** such that all genes in **Γ**_1_ are neighbors to pathway 1, and **Γ**_2_ ∈ **Π** such that all genes in **Γ**_2_ are neighbors to pathway 2, and the set of shared neighbor genes between pathways 1 and 2 is denoted as **Γ**_1∩2_.

When using correlation distance, a pair of genes is considered to be associated if their correlation distance falls below the threshold. By computing the correlation distance of all genes in **Π** to ***χ***_1_ and to ***χ***_2_, we can determine the neighbor gene sets **Γ**_1_ and **Γ**_2_. In this work, we do no compute a distance measure and setting a threshold for forming edges between genes. Instead, we will use elastic-net regression to predict the association between genes in **Π** to gene *a* in pathway ***χ***, by forming an edge between gene *a* and those genes in **Π** that have non-zero coefficient after modeling the expression of *a* with the expressions of genes in **Π**.

To setup the problem, we define **y**_*i*_ as the *T* × 1 expression profile vector of gene *a*_*i*_, *a*_*i*_ ∈ ***χ***, where *T* is the number of samples. Furthermore, **X** is the *T* × ∣**Π**∣ expression profile matrix of genes in **Π**, where ∣**Π**∣ is the number of genes in **Π**, and ***ω***_*i*_ is the *P* × 1 vector of regression coefficients for the elastic-net regression model of *a*_*i*_. With this formulation, we fit the expression profile gene *a*_*i*_ in ***χ*** using the sparse elastic-net regression, with genes in **Π** as predictors. The fitted *P* × 1 coefficient vector ${\hat{{\text{ω}}_{i}}}^{EN}$ will have *N* ≪ *P* nonzero coefficients, and the corresponding genes in **Π** are defined as neighbor genes to gene *a*_*i*_.

To obtain all the neighbor genes of a pathway,

**Data**: ***X***: predictor gene expression matrix, **y**_*i*_: gene expression vector for gene *a*_*i*_ ∈ ***χ***

**for** i = 1 **to** ∣***χ***∣ **do**

 1. Estimate ${\hat{{\text{ω}}_{i}}}^{EN}=arg\;min_{{\text{ω}}_{i}}\left({\left\|{\text{y}}_{i}-\text{X}{\text{ω}}_{i}\right\|}^{2}+\left(1-\lambda \right){\left\|{\text{ω}}_{i}\right\|}^{2}+\lambda {\left\|{\text{ω}}_{i}\right\|}_{1}\right)$;

 2. Select the set of genes, **Γ** ^*i*^, with non-zero coefficients in ${\hat{{\text{ω}}_{i}}}^{EN}$;

**end**

Find $\text{Γ}\;=(\cup_{\text{i}=1}^{|\text{χ}|}{\text{Γ}}^{\text{i}})$, the set of all neighbor genes to ***χ***;

**Algorithm 1**: Neighbor genes discovery

Then, to obtain the shared neighbor genes between pathways 1 and 2,

**Data**: ***X***: predictor gene expression matrix, **y**_*i*_: gene expression vector for gene *a*_*i*_ ∈ ***χ***_1_, **z**_*j*_: gene expression vector for gene *b*_*j*_ ∈ ***χ***_2_

Find **Γ**_1_, the set of all neighbor genes to ***χ***_1_, using Algorithm 1;

Find **Γ**_2_, the set of all neighbor genes to ***χ***_2_, using Algorithm 1;

Find the shared neighbor genes for pathways 1 and 2: **Γ**_1∩2_ = **Γ**_1_∩**Γ**_2_;

**Algorithm 2**: Shared neighbor genes discovery

**Fig 1:**
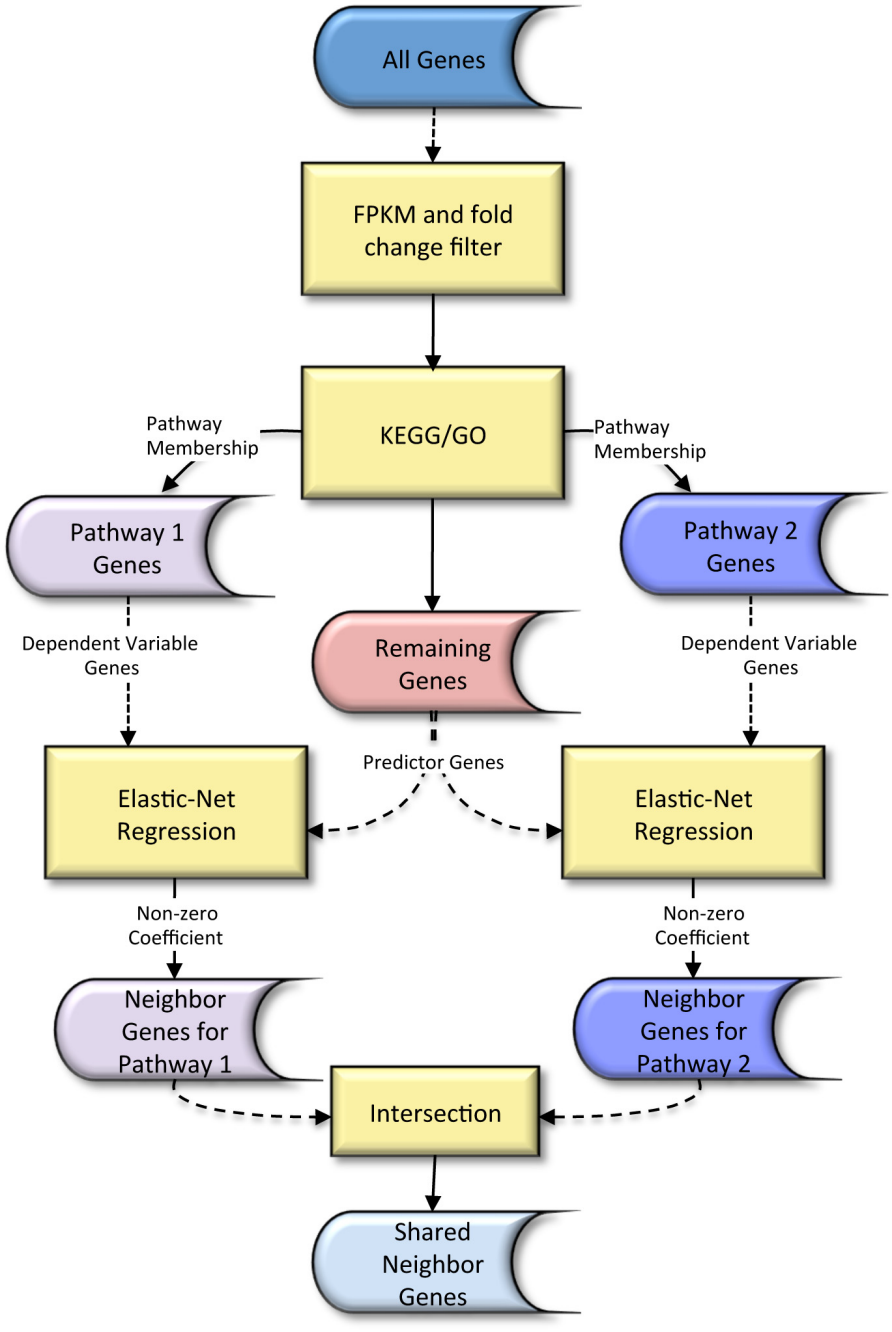
Schematic diagram for finding shared neighbor genes between pathways 1 and 2.

Each predictor gene in **Γ**_1∩2_ has non-zero coefficient for at least one gene in each of the two pathways. Since the gene expressions of these shared neighbor genes can be used to predict the expression of genes in the two pathways in the elastic-net regression model, we hypothesize that they are also good candidates as genes that link together the two pathways. Fig 1 illustrates the steps in Algorithm 1 for finding shared neighbor genes between pathways 1 and 2.

When applied to samples from different cell types, the shared neighbor genes found in different cell types for the same pathway pair may be different due to differential expressions. For example, in a gene knockout experiment, shared neighbor genes found only in the wild type sample but not the knockout sample are potentially paths between the two pathways that are affected by the gene knockout. To simplify our notation, we will drop the pathway subscripts and denote **Γ**_W\K_ for some pathway pair as the set of shared neighbor genes found only in the wild type but not knocked out sample, and **Γ**_W∩K_ as the set of shared neighbor genes that are found in both the wild type and the knocked out sample.

Through gene set enrichment analysis, we can find if any functional overrepresentation and statistical significance exist in the shared neighbor genes that exist under only a specific condition. In particular, we are interested in finding if certain shared neighbor genes exist only in the wild type sample, but not in the knockout sample. Such genes may have a role in connecting the two pathways and their functions are affected by the gene knockout in the upstream pathway. We will then compare the statistically significant overrepresented functions of these genes to known research findings to confirm the role of these genes in connecting the pathways.

## Results

### Mouse Dendritic Cell Knock-out Time-course Data

We applied the proposed method to a time-series mouse dendritic cell RNA-Seq experiment with wild type, MyD88 knockout, and TRIF knockout (KO) cell types. Since both of these adaptor proteins are key components of the TLR signaling pathway, we used the proposed method on each cell type to find shared neighbor genes between TLR signaling pathway and downstream pathways that are affected by immune response caused by LPS stimulation. We then found the set difference gene lists between the wild type and one of the knockout data to find neighbor genes that are unique to each cell type. For elastic-net regression, we used the glmnet implementation in R [32] with λ = 0.5 and fitted the model to explain 75% of the variance in each gene’s time-series expression. For WGCNA, we set soft power to 8 for the adjacency computation.

From previous research, after knocking out MyD88 or TRIF in dendritic cells, we expected to observe changes in expressions in genes belonging to the downstream antigen processing and presentation, apoptosis, and Jak-Stat pathways [17, 33, 34]. We constructed the gene lists for these pathways by obtaining their gene lists from KEGG [1, 2] and AmiGO [35]. For each of the TLR-signaling—antigen procession and presentation, TLR-signaling—apoptosis, and TLR-signaling—Jak-Stat pathway pairs, we used genes not in the two pathways as predictor genes in elastic-net regression.

**Fig 2:**
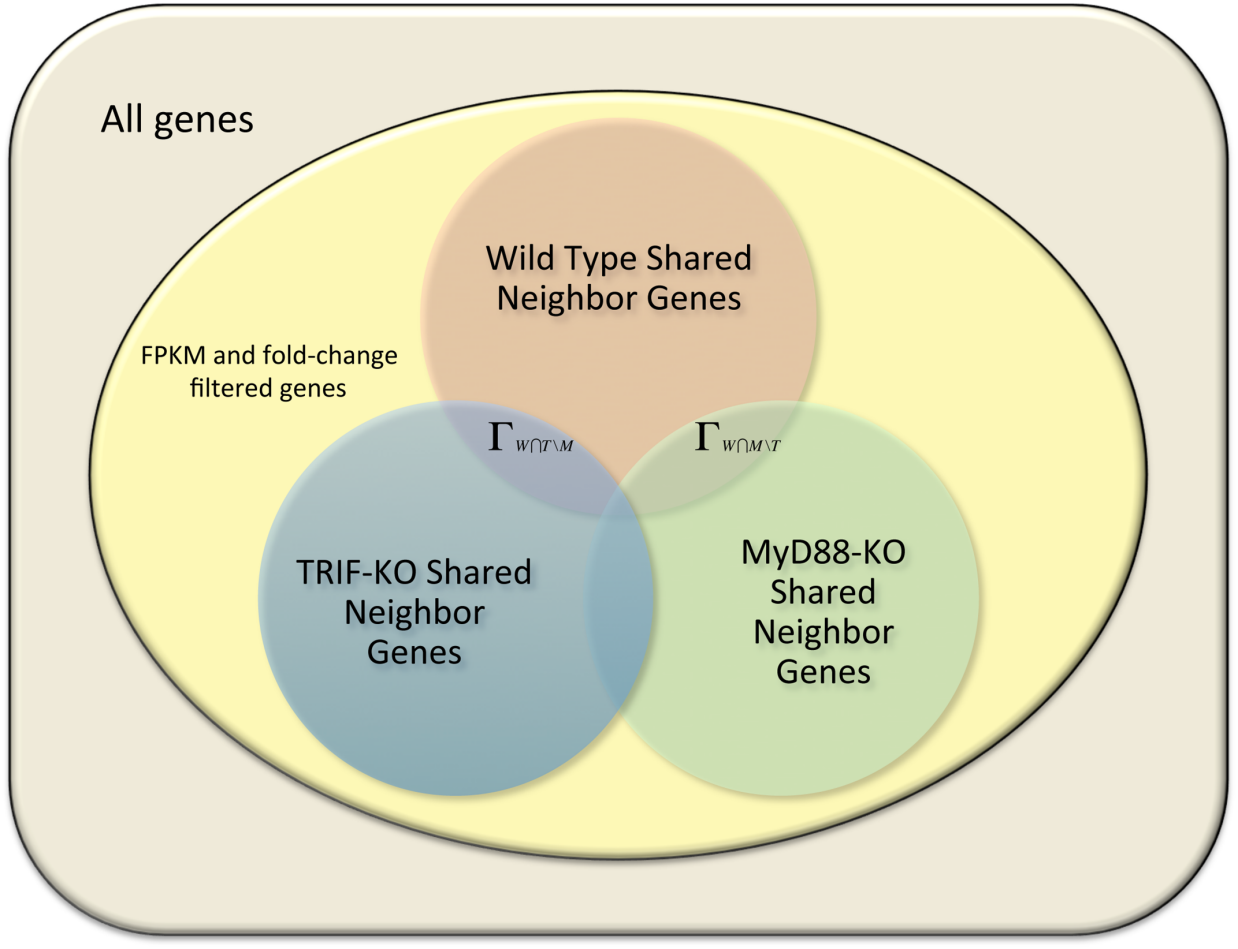
Venn diagram of shared neighbor genes from wild type, MyD88-KO, and TRIF-KO samples.

In the following discussion, we compared the shared neighbor genes in wild type to those in MyD88 KO and TRIF KO cell types to find potential links between TLR signaling pathway and a downstream pathway, and observed which of these links are affected by the gene knockouts. We denote **Γ**_W∩M\T_ as shared neighbor genes that exist between two pathways in both the wild type and MyD88-KO samples, but not in the TRIF-KO sample. This set represents those genes that are associated with the TRIF-dependent part of the TLR-signaling pathway, and their functions are affected by the TRIF-KO. Similarly, **Γ**_W∩T\M_ denotes those shared neighbor genes that exist in both the wild type and the TRIF-KO samples, but not the MyD88-KO sample. Fig 2 shows a Venn diagram of the two gene sets. In our comparisons we used Weighted Correlation Network Analysis (WGCNA) as well as a correlation distance implementation using *μ* = 0.1 as the threshold for finding neighbor genes. We then used gene ontology overrepresentation analysis on these gene set difference lists to find important functions between the pathways that are disabled by the knockout, which knocked out path are these functions associated with, and confirmed our findings through prior research.

#### Antigen processing and presentation

Dendritic cells are critical to the adaptive immune mechanism by acting as an initiator for activating T cells and initiating primary and memory immune responses [36], and presentation of pathogens is accomplished through major histocompatibility complex (MHC) class I or MHC class II molecules. It is well known that when dendritic cells are stimulated by LPS, the downstream antigen processing and presentation functions are activated through the upstream TLR-signaling pathway [37].

We used the proposed method to find shared neighbor genes between TLR signaling pathway and antigen processing and presentation for each of the wild type, MyD88 knockout, and TRIF knockout cell types, denoted as **Γ**_W_, **Γ**_M_, and **Γ**_T_, respectively. We then constructed the sets **Γ**_W∩T\M_ and **Γ**_W∩M\T_ as described in the Materials and Methods Section. The same steps were followed for correlation distance and WGCNA. The corresponding set difference gene sets using correlation distances are denoted as **Θ**_W∩T\M_ and **Θ**_W∩M\T_, and those for WGCNA are denoted as **Φ**_W∩T\M_ and **Φ**_W∩M\T_.

**Fig 3:**
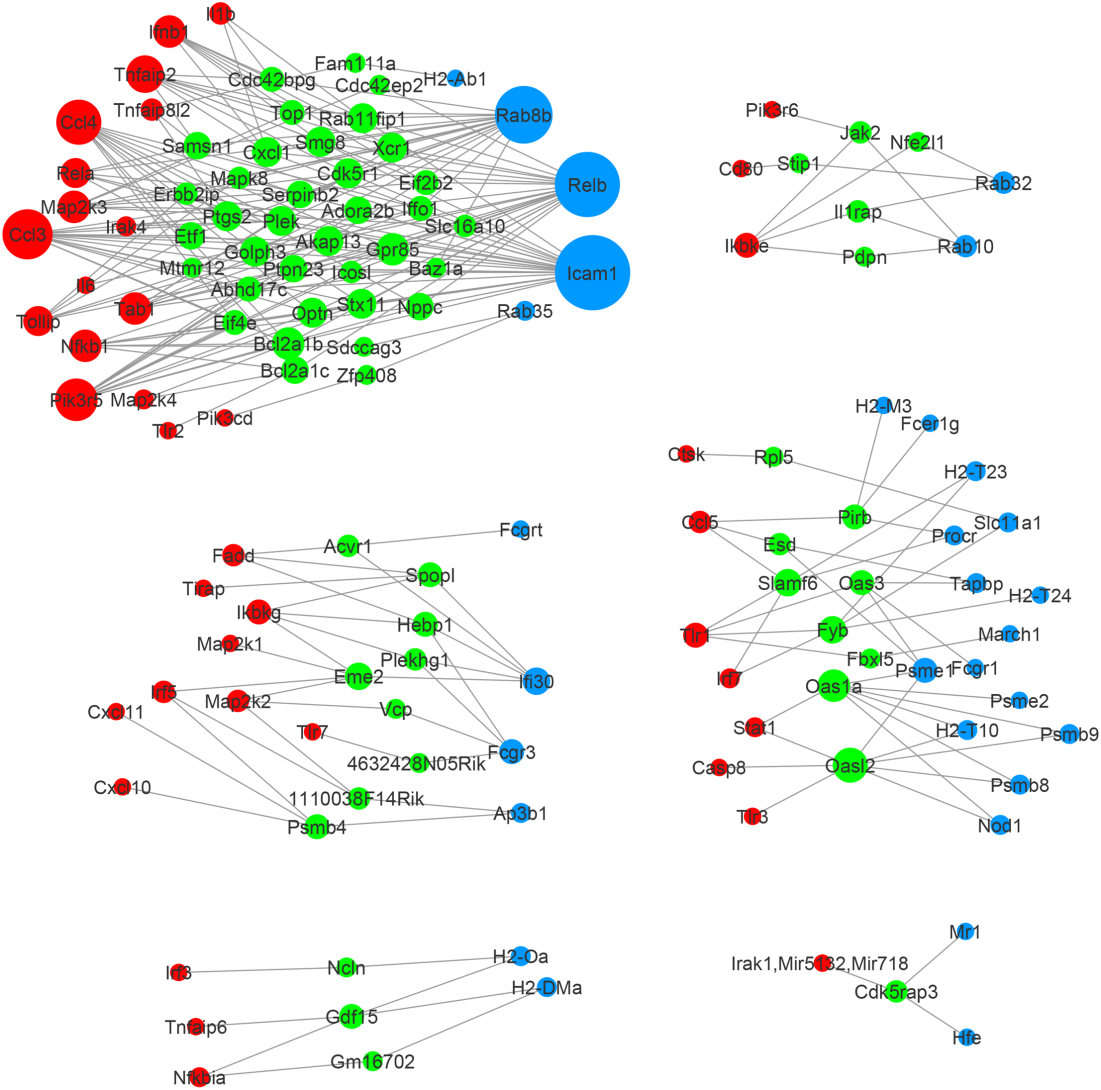
Network edges of shared neighbor genes found in wildtype and TRIF-KO, but not in MyD88-KO, by elastic-net. Red nodes: TLR signaling pathway genes. Blue nodes: Antigen processing and presentation genes. Green nodes: Shared neighbor genes.

**Fig 4:**
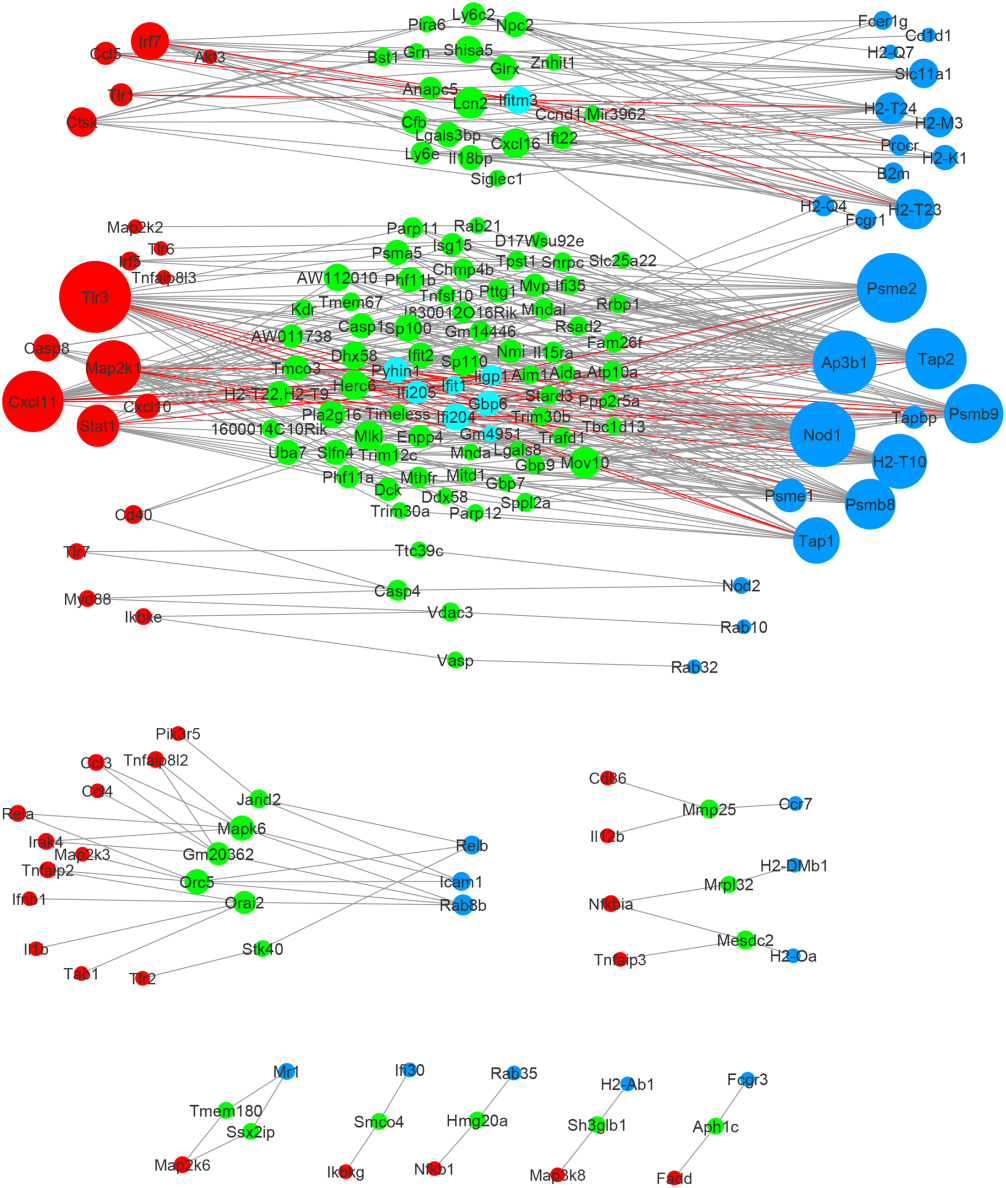
Network edges of shared neighbor genes found in wildtype and MyD88-KO, but not in TRIF-KO, by elastic-net. Red nodes: TLR signaling pathway genes. Blue nodes: Antigen processing and presentation genes. Green nodes: Shared neighbor genes. Cyan nodes: Shared neighbor genes with “reseponse to interferon-*β* GO term. Red edges: edges from cyan genes to pathway genes.

In Figs 3 and 4 we have drawn figures of shared neighbor genes with their connected pathway genes in **Γ**_W∩T\M_ and **Γ**_W∩M\T_ using Cytoscape [38], respectively.

Genes in each of the six sets can be found in Table A in S1 File. For sets **Γ**_W∩T\M_ and **Γ**_W∩M\T_ we found 63 and 108 genes, respectively. For sets **Θ**_W∩T\M_ and **Θ**_W∩M\T_, we found 689 and 276 genes, respectively. For sets **Φ**_W∩T\M_ and **Φ**_W∩M\T_, we found 186 and 285 genes, respectively. The numbers of genes in the two gene sets found by elastic-net are much closer to each other than those found by the correlation distance implementation, where the set with more genes is almost 3 times larger than the smaller one. Compared to the correlation distance implementation, the two gene sets found by WGCNA also have more comparable numbers of genes. This is an advantage for the elastic-net method and WGCNA due to the impact that gene list size has on the significance of terms discovered, making the overrepresentation results for the elastic-net-generated lists much more comparable.

To find overrepresented GO terms in the gene lists we used Gorilla [39] with **Π** as background. The significant gene ontology terms and the genes with those GO terms in **Γ**_W∩T\M_ and **Γ**_W∩M\T_ are listed in Tables 1 and 2, respectively. The top 5 significant gene ontology terms and their FDR for elastic-net, correlation distance, and WGCNA are shown in Table B in S1 File. Of the 6 gene sets, only **Γ**_W∩M\T_ and **Φ**_W∩M\T_ contain statistically significant gene ontology terms related to immune process after multiple testings correction using FDR. In particular, we found that both **Γ**_W∩M\T_ and **Φ**_W∩M\T_ are significantly represented with genes in response to cytokine (FDR = 3.63E-4 for elastic-net and FDR = 1.19E-9 WGCNA) and response to interferon-*β* (FDR = 1.39E-4 for elastic-net and FDR = 2.49E-9 for WGCNA). Both of these gene sets are overrepresented in similar GO terms, with WGCNA having the more significant gene set in terms of FDR, but the elastic-net method also having significant FDR. On the other hand, for the correlation distance approach, while **Θ**_W∩M\T_ also contains genes with the gene ontology term response to interferon-*β*, its FDR is 1E0, with no other terms showing any statistical significance.

**Table 1:** Significant GO terms and genes for **Γ**_W∩T\M_ between TLR Signaling Pathway and Antigen Processing and Presentation.

GO Terms	Genes
positive regulation of determination of dorsal identity	Acvr1, Mapk8
positive regulation of response to wounding	Jak2, Ptgs2, Adcra2b, Plek, Pdpn
positive regulation of phosphatase activity	Jak2, Rpl5, Plek

**Table 2:** Significant GO terms and genes for **Γ**_W∩M\T_ between TLR Signaling Pathway and Antigen Processing and Presentation.

GO Terms	Genes
response to interferon-*β*	Ifi205, Ifit1, Gm4951, Gbp6, Ifitm3, Ifi204, Pyhin1, Iigp1
cellular response to interferon-*β*	Ifi205, Ifit1, Gbp6, Gm4951, Ifi204, Pyhin1, Iigp1
response to cytokine	Gbp7, Gbp6, Gm4951, Pyhin1, Ifit2, Ifi205, Ifit1, Isg15, Ifi204, Ifitm3, Gbp9, Iig1, Cxcl16
response to other organism	Gbp7, Sp110, Gbp6, Casp1, Ddx58, Dhx58, Ifit2, Ifit1, Isg15, Ifitm3, Gbp9, Iigp1, Rsad2, Lcn2
cellular response to cytokine stimulus	Ifi205, Gbp7, Ifit1, Gm4951, Gbp6, Ifi204, Gbp9, Pyhin1, Iig1, Ifit2

Through literature search, we were able to confirm the findings by elastic-net and WGCNA with prior study showing that TRIF is responsible for the induction of interferon-*β* [40], and more recent research showing that the inhibition of interferon-*β* impairs the antigen presentation functions of dendritic cells [41]. Using the proposed methodology, we were able to predict correctly that genes related to the response to interferon-*β* are acting as intermediary between TLR signaling pathway and genes responsible for antigen processing and presentation.

#### Apoptosis

Apoptosis of dendritic cells plays an important role in the balancing of immune tolerance and the development of autoimmunity [42]. An excessive activation of dendritic cells can induce tissue-specific and systemic autoimmune symptoms [42–44], and an environment where significant dendritic cell apoptosis occurs is immunosuppressive [45]. It has been shown that dendritic cell apoptosis is controlled by the TLR4-mediated TRIF-dependent signaling pathway [46] and that Type-1 interferons are necessary and sufficient for the induction of apoptosis for dendritic cells [47]. In particular, interferon-*γ* has been shown to induce nitric oxide synthase in mouse dendritic cells, and the production of nitric oxide is associated with dendritic cell apoptosis [48]. Furthermore, cytokines has been shown to be the path of TRIF-induced apoptosis [49].

For TLR signaling pathway and apoptosis we have found 65 and 63 genes for **Γ**_W∩T\M_ and **Γ**_W∩M\T_, respectively. For **Θ**_W∩T\M_ and **Θ**_W∩M\T_ we found 842 and 337 genes, respectively. For **Φ**_W∩T\M_ and **Φ**_W∩M\T_ we found 201 and 274 genes, respectively. The genes for each gene set are listed in Table A in S1 File. Again, the numbers of genes found are much more comparable in the elastic-net implementation and WGCNA, with elastic-net finding almost exactly the same number of genes in the two shared neighbor gene sets.

We analyzed the gene sets for overrepresented GO terms, and the top 5 significant gene ontology terms for each are shown in Table C in S1 File. The significant gene ontology terms and the genes with those GO terms for **Γ**_W∩T\M_ and **Γ**_W∩M\T_ are listed in Tables 3 and 4, respectively. For **Γ**_W∩M\T_, we found that response to interferon-*γ* is significantly overrepresented with FDR = 1.15E-2, and no immune related terms were found to be significant in the **Γ**_W∩T\M_ set. Response to interferon-*γ* was also found to be significant in **Φ**_W∩M\T_ with FDR = 8.75E-4. Again, both elastic-net and WGCNA found similar overrepresented GO terms, with WGCNA having more significant FDR values.

**Table 3:** Significant GO terms and genes for **Γ**_W∩T\M_ between TLR Signaling Pathway and Apoptosis.

GO Terms	Genes
protein dephosphorylation	Ptpre, Ppp1r15b, Ptpn23, Dusp16, Fbxw11, Ssh2
dephosphorylation	Ptpre, Ppp1r15b, Ptpn23, Dusp16, Fbxw11, Ssh2, Mtmr14
ubiquitin-dep. protein catabolic proc.	Ptpn23, Usp16, Siah2, Psmb7, Rffl, Ube4a, Fbxw11
proteolysis	Bcl10, Siah2, Ptpn23, Usp16, Psmb7 Rffl, Ndel1, Plat, Ube4a, Fbxw11, Metap1
positive reg. of platelet activation	Plek, Pdpn

**Table 4:** Significant GO terms and genes for **Γ**_W∩M\T_ between TLR Signaling Pathway and Apoptosis.

GO Terms	Genes
cellular response to cytokine stimulus	Gbp7, Gm4951, Gbp6, Ifi204, Gbp9 Gbp3, Iigp1, Ifit2
response to protozoan	Gbp7, Gbp6, Gbp9, Gbp3, Iigp1
cellular response to interferon-*β*	Gbp6, Gm4951, Ifi204, Gbp3, Iigp1
cellular response to interferon-*γ*	Gbp7, Gbp6, Gbp9, Gbp3

For correlation distance, while **Θ**_W∩M\T_ contains several highly significant immune related GO terms, it is significantly overrepresented only in response to interferon-*β* (FDR = 1.65E-4), but not interferon-*γ*. In this case, both the elastic-net implementation and WGCNA were again able to find result that is supported by existing studies. While the correlation distance implementation was also able to find that significant immune related processes are altered by TRIF-KO, it’s results are not as precise and accurate.

#### Jak-Stat signaling pathway

The Jak-Stat signaling pathway is responsible for signal transduction in development and homeostasis in animals, and is the primary signal mechanism for cytokines [50]. It has been pointed out that because the Jak-Stat signaling pathway is downstream of interferon-*β* production, it should be affected by the TRIF-dependent pathway [51], but not the MyD88-dependent pathway [52].

The genes in **Γ**_W∩T\M_, **Γ**_W∩M\T_, **Θ**_W∩T\M_, and **Θ**_W∩M\T_ are listed in Table A in S1 File. For sets **Γ**_W∩T\M_ and **Γ**_W∩M\T_ we have found 137 and 114 genes, respectively. For sets **Θ**_W∩T\M_ and **Θ**_W∩M\T_ we found 734 and 308 genes, respectively. For sets **Φ**_W∩T\M_ and **Φ**_W∩M\T_ we found 378 and 407 genes, respectively.

The top significant gene ontology terms for each gene set are shown in Table D in S1 File. The significant gene ontology terms and the genes with those GO terms for **Γ**_W∩T\M_ and **Γ**_W∩M\T_ are listed in Tables 5 and 6, respectively. For TLR signaling Jak-Stat pathways, both the elastic-net implementation and WGCNA discovered in **Γ**_W∩M\T_ and in **Φ**_W∩M\T_ genes that are overrepresented with the cellular response to interferon-*β* term (FDR = 3.48E-5 and 9.56E-9, respectively), and found no significant functional overrepresentation related to immune process present in **Γ**_W∩T\M_ nor **Φ**_W∩T\M_, in agreement with existing literature. On the other hand, the gene sets obtained from correlation distance implementation also did not find any GO terms with significant functional overrepresentation. For **Γ**_W∩T\M_, gene ontology analysis did find several terms relating to antigen processing and presentation, but the lowest FDR of these terms is only 6.16E-1.

**Table 5:** Significant GO terms and genes for **Γ**_W∩T\M_ between TLR Signaling and Jak-Stat Pathways.

GO Terms	Genes
positive regulation of cell cycle	Cdk5r1, Bcl2l11, Vps4b, Hspa2, Igf1r Tnf, E2f8, Eif4e, Nr4a3, Mad2l1
NIK/NF-*κ*B signaling	Nfkb2, P49/p100, Relb, Rel

**Table 6:** Significant GO terms and genes for **Γ**_W∩M\T_ between TLR Signaling and Jak-Stat Pathways.

GO Terms	Genes
cellular response to interferon-*β*	Ifi205, Ifit1, Gbp6, Gm4951, Ifi204 Gbp3, Iigp1, Gbp2
response to cytokine	Ifi205, Gbp7, Ifit1, Gm4951, Gbp6, Isgl1, Ifi204, Trim56, Ptgs2, Gbp3 Gbp3, Iigp1, Gbp2
multi-organism process	Sp110, Gbp7, Gbp6, Chmp4b, Trim56, Hdac1 Ifih1, Gbp3, Ddx58, Ccnk, Gbp2, Rsad2 Tap2, Ifit1, Isg15, Ddx60, Iigp1
defense response to other organism	Gbp7, Ifit1, Gbp6, Isg15, Trim56, Ifih1 Ddx60, Gbp3, Iigp1, Ddx58, Gbp2, Rsad2

### Caucasian RNA-Seq Data

With the Caucasian RNA-Seq datasets, we again applied the elastic-net, correlation distance, and WGCNA to discover shared neighbor genes between TLR signaling pathway and antigen processing and presentation, apoptosis, and Jak-Stat pathways. We used the same parameters as used in the mouse dendritic cells analysis. Since there are no gene knockouts in this experiment, we only compared the shared neighbor gene sets found by elastic-net, correlation distance, and WGCNA in the wild type samples, denoted as **Γ**, **Θ**, and **Φ**, respectively. The gene sets are listed in Table A in S2 File, and the top overrepresented GO terms for shared neighbor genes between TLR signaling pathway and antigen processing and presentation, apoptosis, and Jak-Stat pathways are listed in Tables B, C, and D in S2 File, respectively.

Elastic-net, correlation distance, and WGCNA found 82, 42, and 173 shared neighbor genes between TLR signaling pathway and antigen processing and presentation. In Table B in S2 File, we can see that elastic net found that type I interferon signaling pathway and response to type I interferon to be significantly overrepresented with p-values 4.20E-4 and 5.53E-4, respectively. While specific terms related to interferon-*β* was not found to be overrepresented, it is a member of the human type I interferon family. On the other hand, for correlation distance and WGCNA, no immune process related GO terms were found to be overrepresented. The top terms for both are populated with metabolic and biosynthetic processes terms.

For apoptosis, 110, 142, and 794 shared neighbor genes were found by elastic-net, correlation distance, and WGCNA, respectively. From GO analysis, we can see that correlation distance and WGCNA found no overrepresented immune related GO terms, while elastic-net found lymphocyte and leukocyte mediated immunity to be overrepresented, which include genes such as FCER2 (CD23), RasGRP1, and HLA-E. From literature we know that CD23 is induced by TLR4 [53], and that high apoptotic rates are often correlated to the expression of CD23 [54]. RasGRP1 is a guanine nucleotide exchange factor whose expression is upregulated by LPS and other TLR agonists [55], and promotes B cell receptor-induced apoptosis [56]. Furthermore, the transcription of HLA-E, the human major histocompatibility complex (MHC) class Ib gene, is shown to be mediated by interferon-*γ* [57], and is known to elicit apoptosis in natural killer cells [58].

For Jak-Stat pathway, 122, 95, and 268 shared neighbor genes were found by elastic-net, correlation distance, and WGCNA, respectively. In this case, we see that none of the shared neighbor gene sets are overrepresented in immune-related GO terms. Shared neighbor gene set found by elastic-net (**Γ**) is enriched in phosphorylation and metabolic processes, whereas **Θ** and **Φ** are enriched with GO terms related to transcription and RNA synthesis.

These results show that with the Caucasian RNA-Seq data, the proposed elastic-net approach is more sensitive than WGCNA and correlation distance. Since these Caucasian individual samples had not been stimulated with LPS, the immune response pathways are not expected to be consistently activated across the samples. Therefore, any relationship the upstream TLR signaling pathway has with a downstream pathway, and with any intermediary genes will be weak, making the inference of gene-gene interactions a difficult task. In this regard, while the significances are not high after correcting for multiple testing, several immune process related GO terms with significant p-values were found by elastic-net for antigen processing and presentation and for apoptosis. In the case of apoptosis, several genes are shown by previous research to be related to both the TLR signaling pathway and apoptosis, despite the fact that the GO terms are not known to be be their intermediary. These results show that elastic-net can be more sensitive in detecting gene-to-gene relationships when the signals are weak.

## Conclusion

The discovery of genes that link together the activities of different pathways are crucial to the advance of pathway analysis and systems biology. Here we have proposed a methodology using sparse regression approach to specifically discover genes that are shared neighbors of two different pathways. We have also shown that the method chosen to select neighbor genes can greatly affect the outcome of the subsequent overrepresentation analysis. In this paper, we have adopted the elastic-net regression approach and select those predictor genes with non-zero coefficients as neighbors to the gene whose expression was modeled. We show in a series of comparisons that the elastic-net implementation can predict gene-to-gene relationships enriched with comparable GO terms to those predicted by WGCNA. At the same time, elastic-net is shown to be more sensitive than WGCNA in datasets that contains only weak relationships between genes, and is superior to a simple correlation distance implementation in all cases tested.

It should be noted that while it is possible that a gene in one pathway studied in our work may interact directly with a gene in the other pathway, we only attempt to find genes outside of the two pathways, and whose expression profiles show interactions with both pathways. In order to find direct interactions, when modeling genes in pathway 1, we can add genes from pathway 2 to the rest of the genes, and those genes in pathway 2 with non-zero coefficients can then be linked directly to pathway 1, and vice versa. So far we have used only the non-zero coefficient requirement as a selection criteria for neighbor genes of those in the two pathways. One future direction for the development of this method is to introduce a more sophisticated selection criteria to more precisely select neighbor genes. One such approach can be the use of the coefficient values as a criteria, where we can rank the predictor genes with non-zero coefficients and select only the top genes as neighbors. Or we could compute the geometric mean of the number of times a predictor is non-zero for the two pathways. Furthermore, the gene selection can be further validated by having p-values for the fitted coefficients in elastic-net. While currently there is no known approach for computing the p-value for the coefficients in elastic-net, one may try to do a large number of permutations of the gene expressions of the dependent variable gene, and see how often a selected gene from the original is selected in the permutations.

## Supporting Information

S1 File Significant genes and GO terms found by elastic-net, WGCNA and correlation distance for time-course mouse dendritic cell RNA-Seq data. Complete gene lists of **Γ**_W∩T\M_, **Γ**_W∩M\T_, **Θ**_W∩T\M_, **Θ**_W∩M\T_, **Φ**_W∩T\M_, and **Φ**_W∩M\T_ (**Table A**). Top GO terms for shared neighbor genes between TLR-signaling pathway and antigen processing and presentation genes (**Table B**). Top GO terms for shared neighbor genes betwen TLR-signaling pathway and apoptosis genes (**Table C**). Top GO terms for shared neighbor genes between TLR-signaling pathway and Jak-Stat pathway genes (**Table D**).
(XLSX)

S2 File Significant genes and GO terms found by elastic-net, WGCNA and correlation distance for HapMap Caucasian individual RNA-Seq data. Complete gene lists of **Γ**, **Θ**, and **Φ** (**Table A**). Top GO terms for shared neighbor genes between TLR-Signaling pathway and antigen processing and presentation genes (**Table B**). Top GO terms for shared neighbor genes between TLR-Signaling pathway and apoptosis genes (**Table C**). Top GO terms for shared neighbor genes between TLR-Signaling pathway and Jak-Stat pathway genes (**Table D**).
(XLSX)

S1 Fig Per-base quality scores and mapping-rate for all samples. (PDF)
