# Supplementary material for: Discovery of Intermediary Genes between Pathways Using Sparse Regression
Source: PLoS One. 2015 Sep 8;10(9):e0137222. doi: 10.1371/journal.pone.0137222 (PMC4562633; doi:10.1371/journal.pone.0137222)
Supplement: S1 Fig — (PDF) [file pone.0137222.s003.pdf]

Quanlity scores for all RNA-Seq samples

Wildtype mouse dendritic cell at t=0h before stimulation by LPS

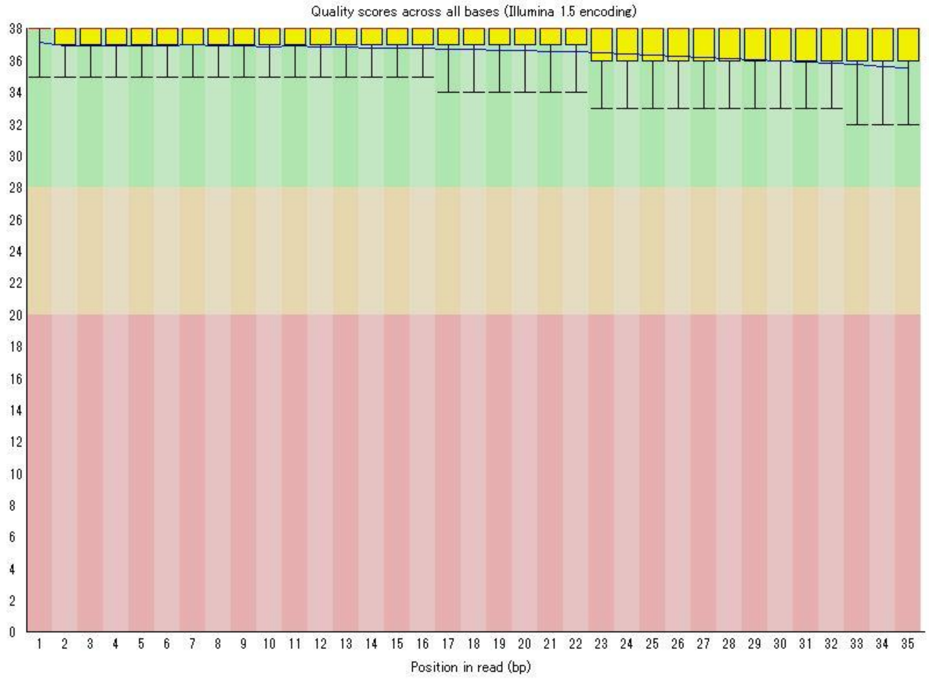

Wildtype mouse dendritic cell at t=0.5h after stimulation by LPS

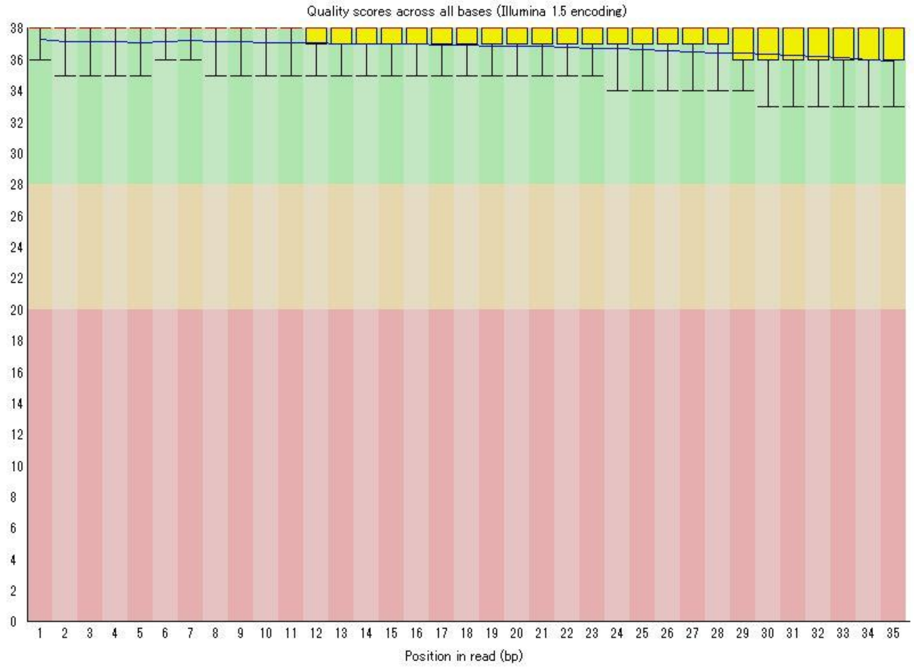

Wildtype mouse dendritic cell at t=1h after stimulation by LPS

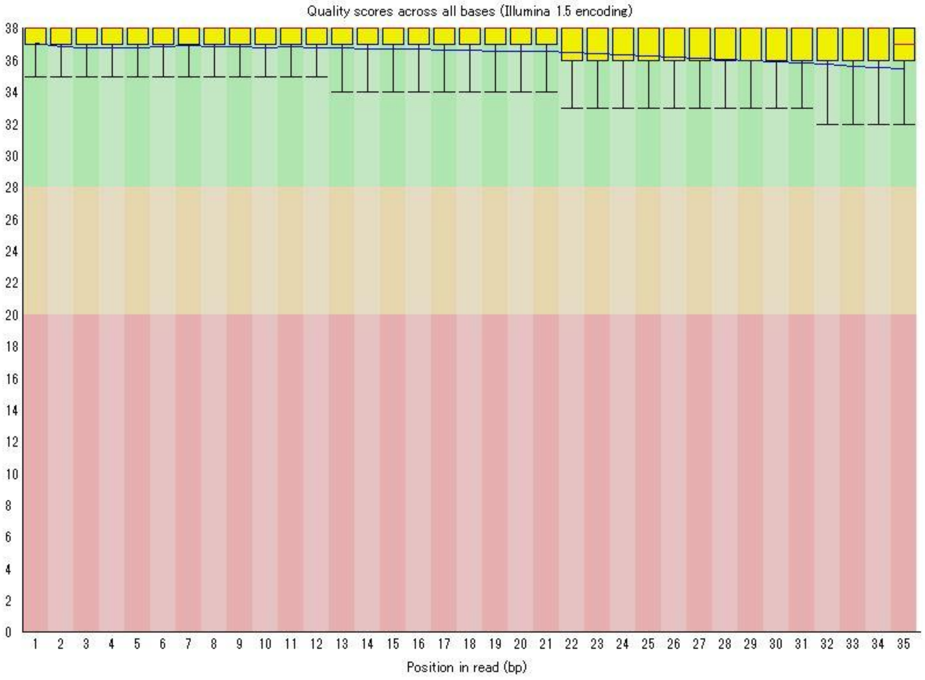

Wildtype mouse dendritic cell at t=2h after stimulation by LPS

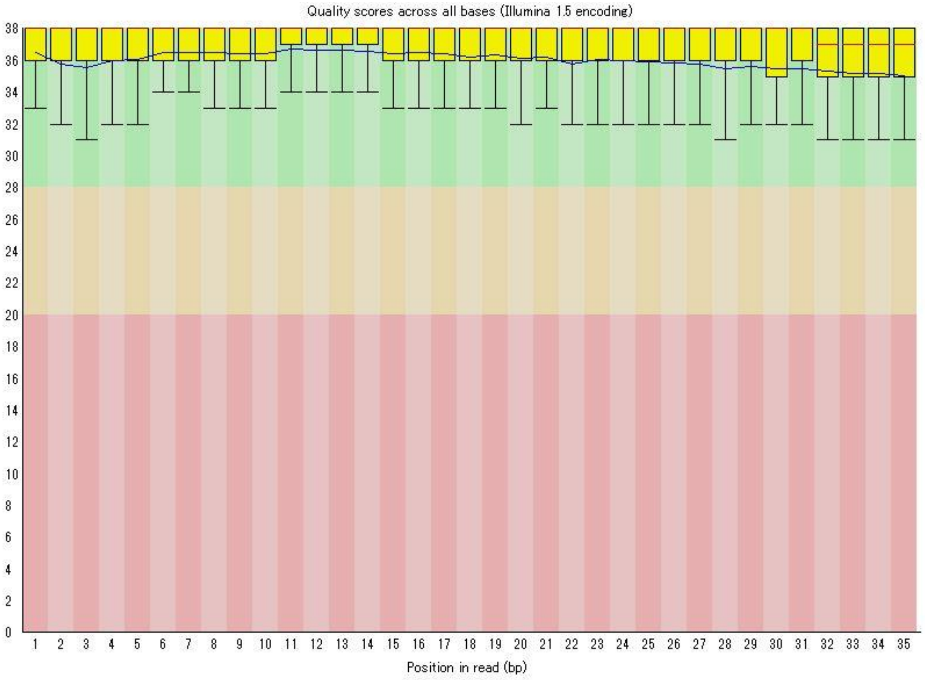

Wildtype mouse dendritic cell at t=3h after stimulation by LPS

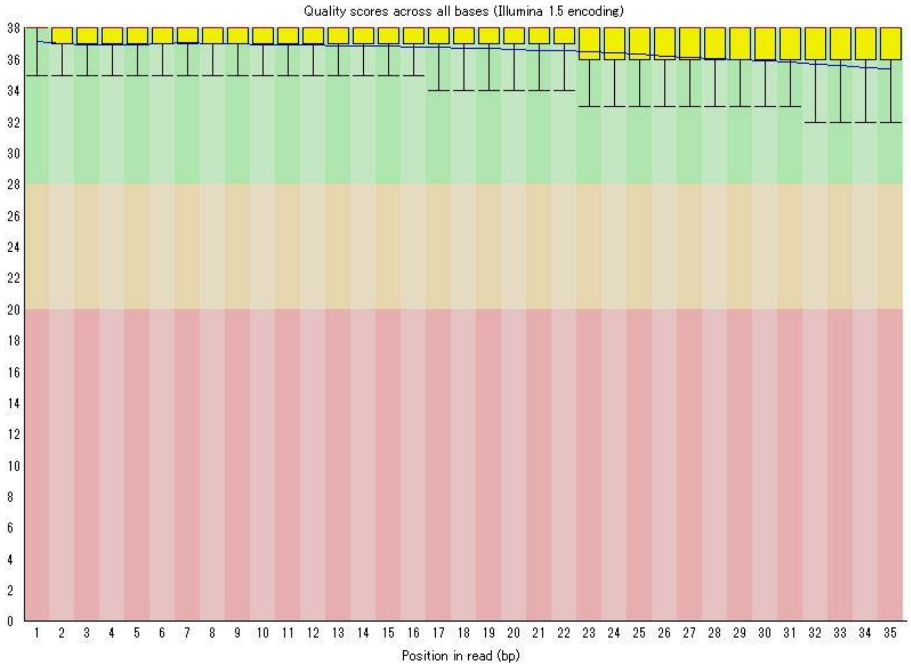

Wildtype mouse dendritic cell at t=4h after stimulation by LPS

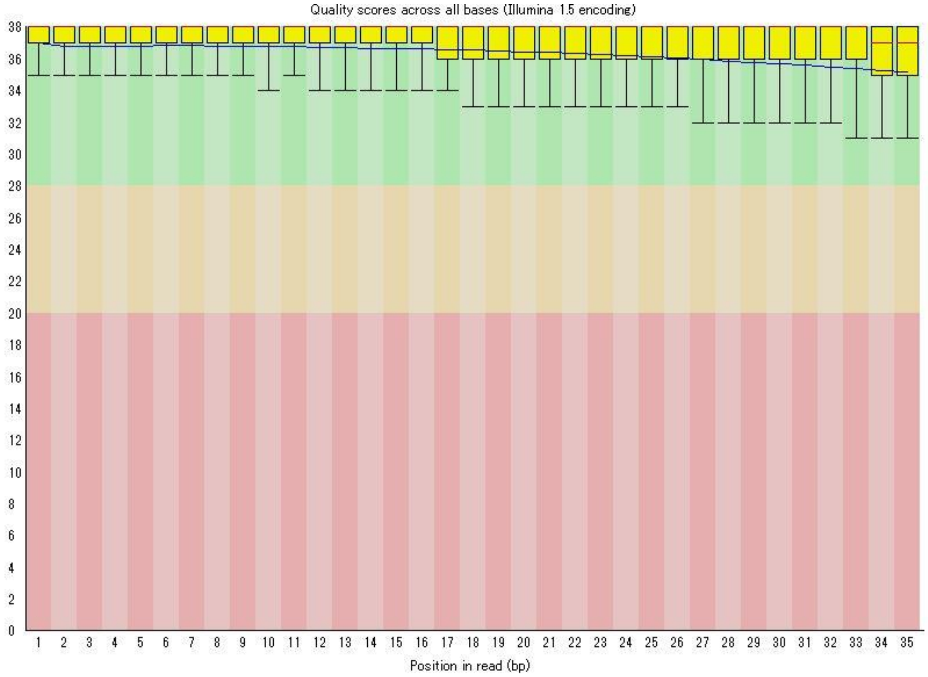

Wildtype mouse dendritic cell at t=6h after stimulation by LPS

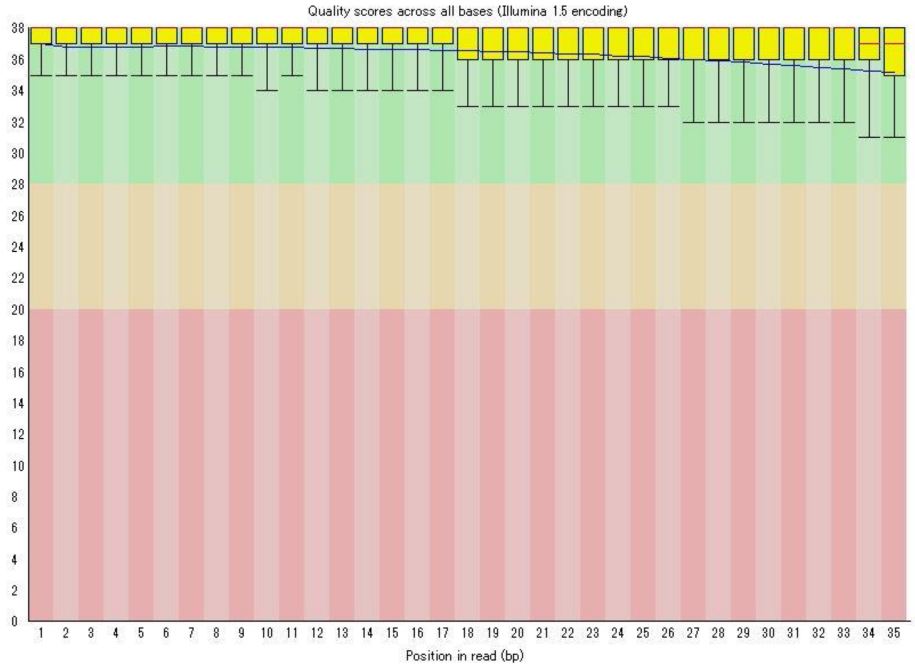

Wildtype mouse dendritic cell at t=8h after stimulation by LPS

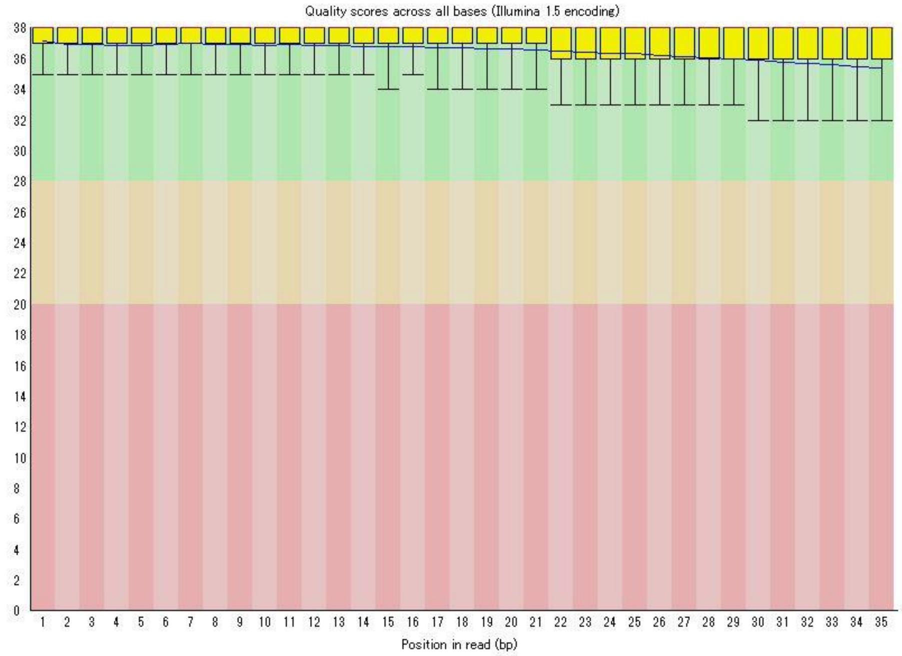

Wildtype mouse dendritic cell at t=16h after stimulation by LPS

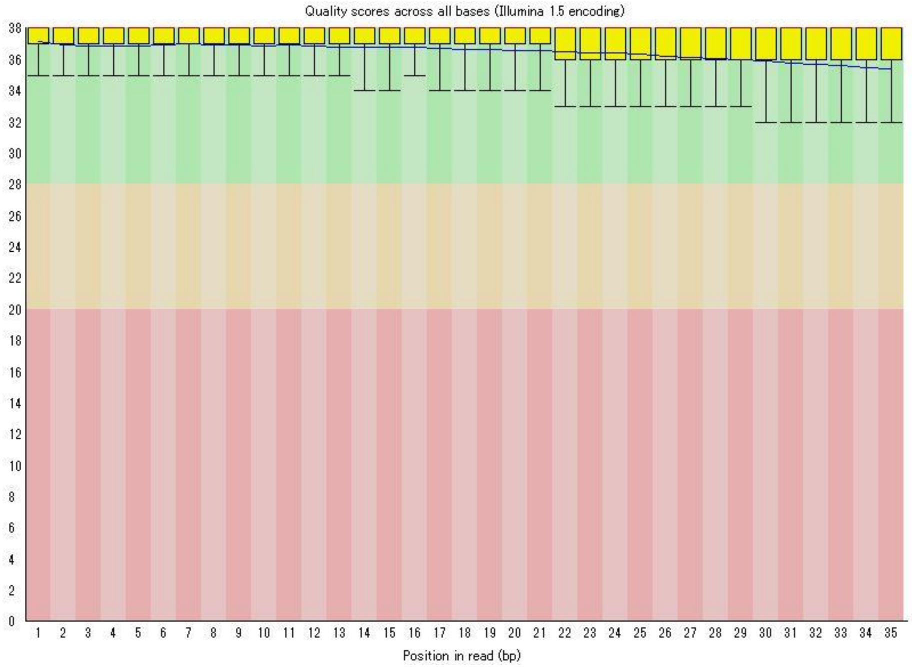

Wildtype mouse dendritic cell at t=24h after stimulation by LPS

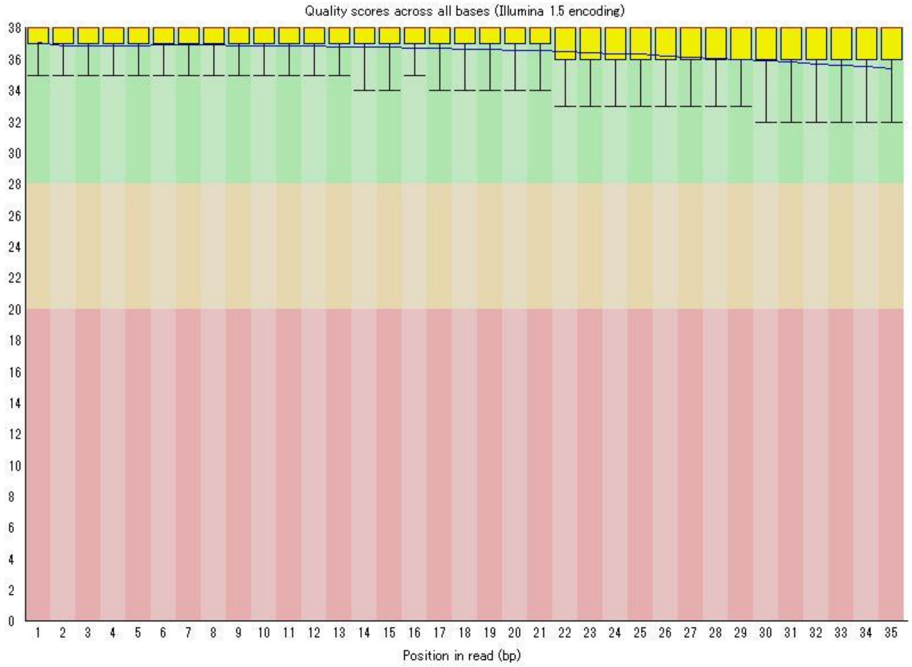

MyD88-Knockout mouse dendritic cell at t=0h before stimulation by LPS

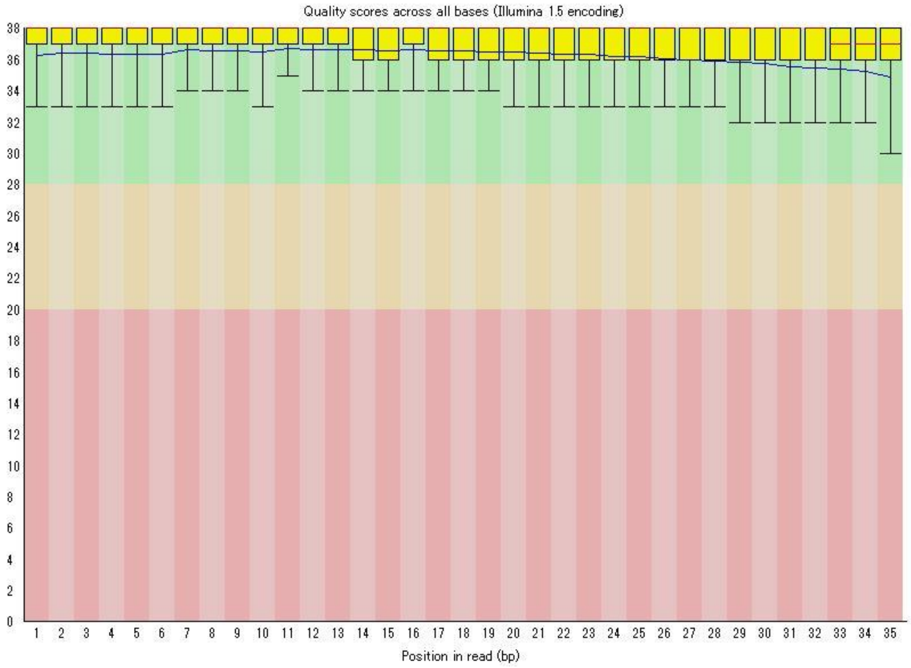

MyD88-Knockout mouse dendritic cell at t=0.5h after stimulation by LPS

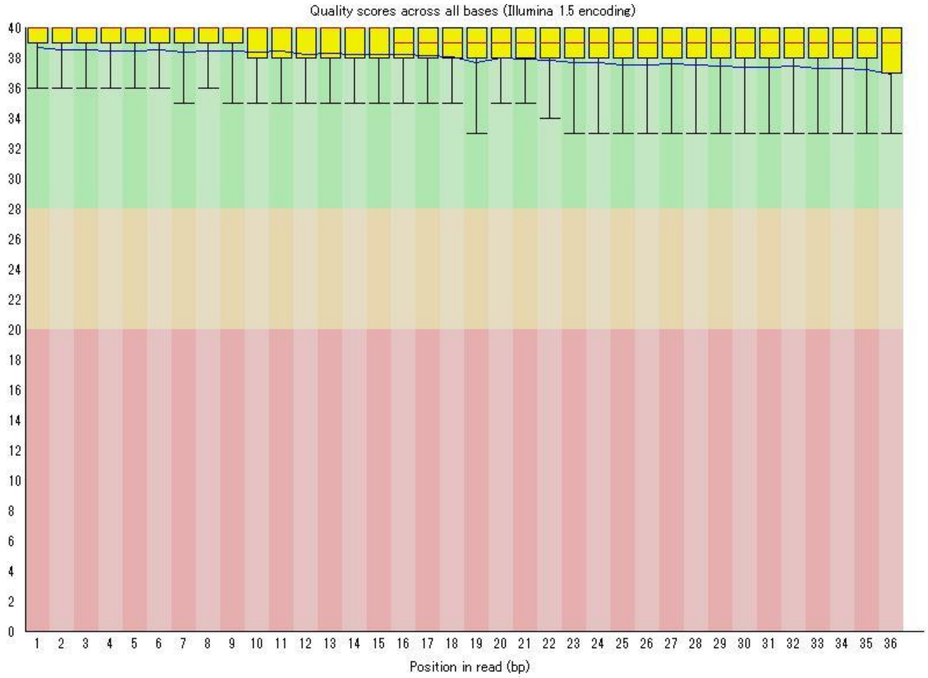

MyD88-Knockout mouse dendritic cell at t=1h after stimulation by LPS

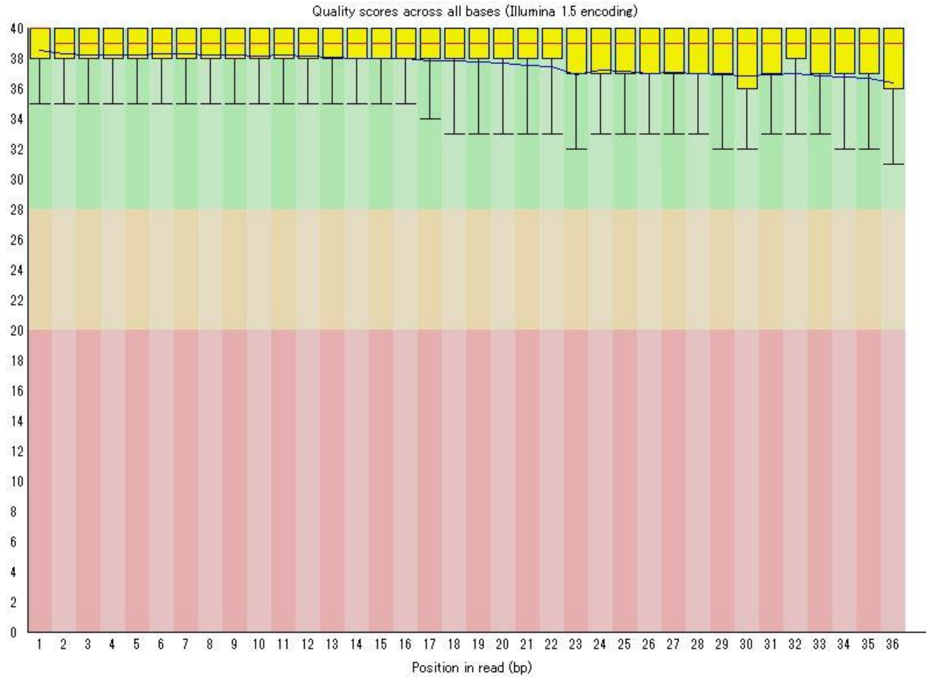

MyD88-Knockout mouse dendritic cell at t=2h after stimulation by LPS

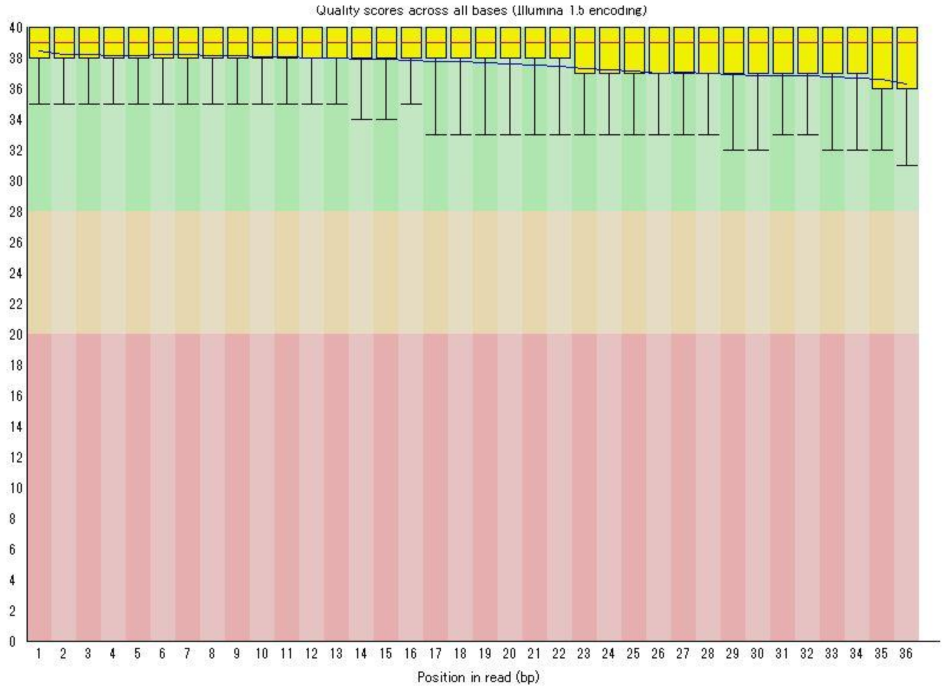

MyD88-Knockout mouse dendritic cell at t=3h after stimulation by LPS

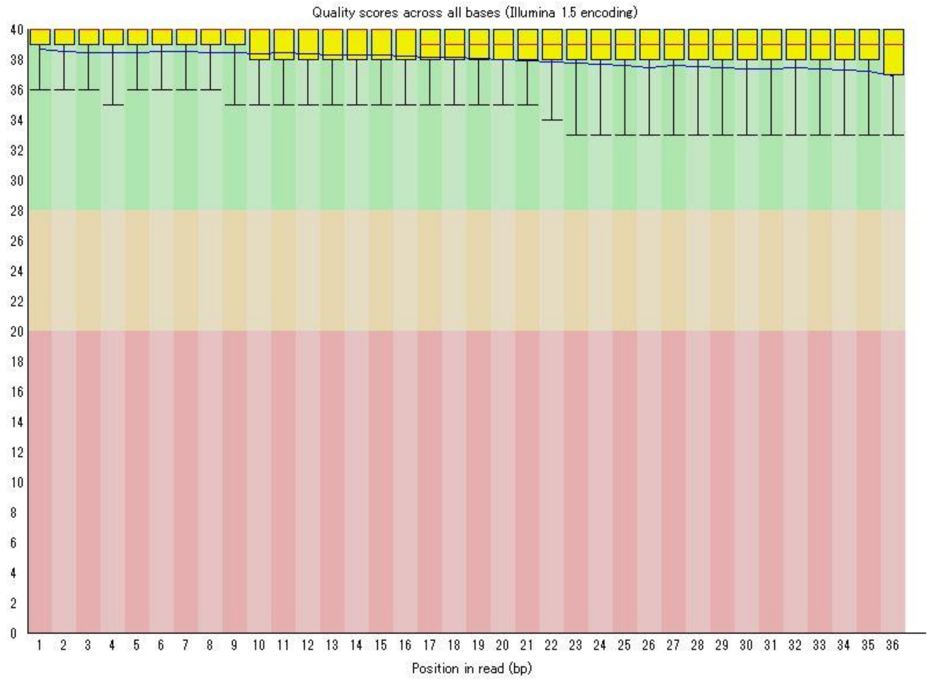

MyD88-Knockout mouse dendritic cell at t=4h after stimulation by LPS

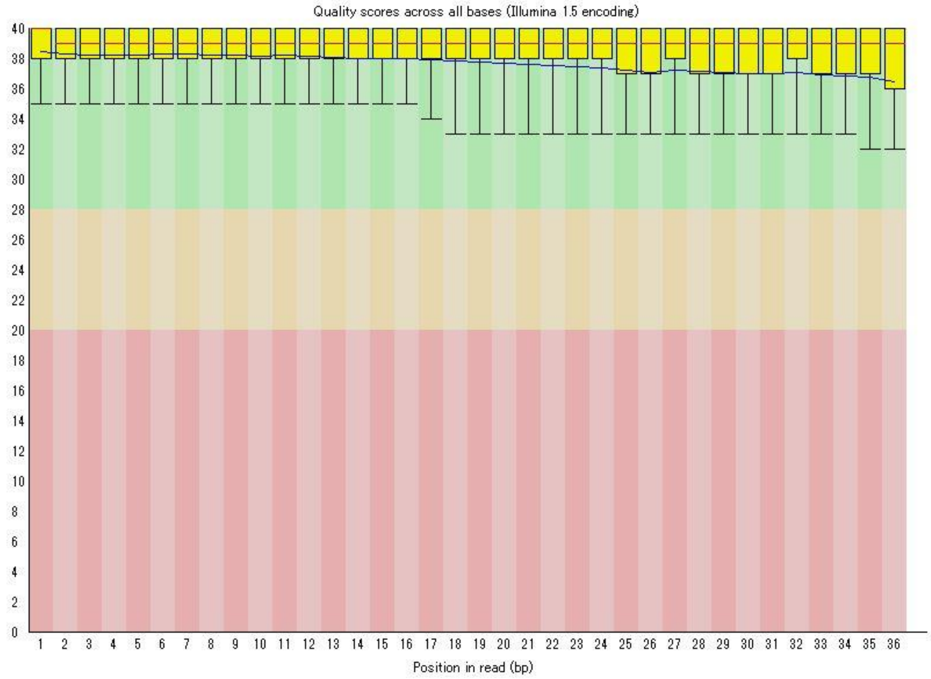

MyD88-Knockout mouse dendritic cell at t=6h after stimulation by LPS

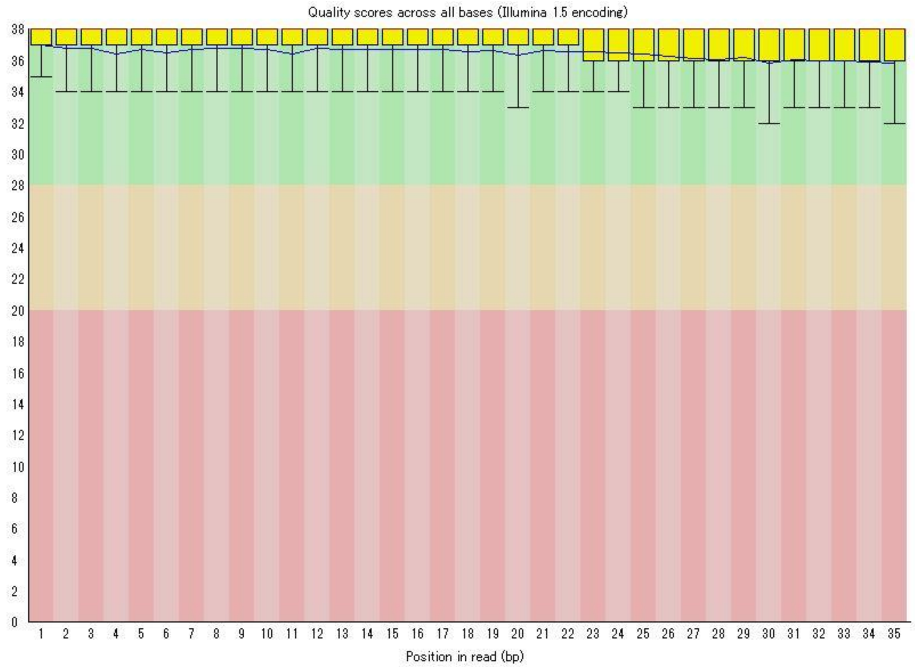

MyD88-Knockout mouse dendritic cell at t=8h after stimulation by LPS

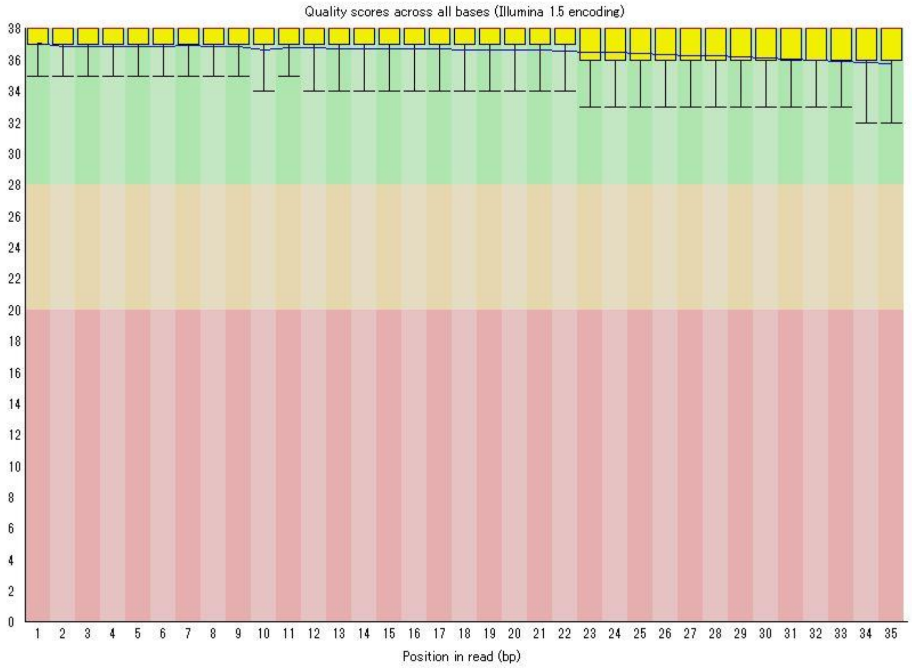

MyD88-Knockout mouse dendritic cell at t=16h after stimulation by LPS

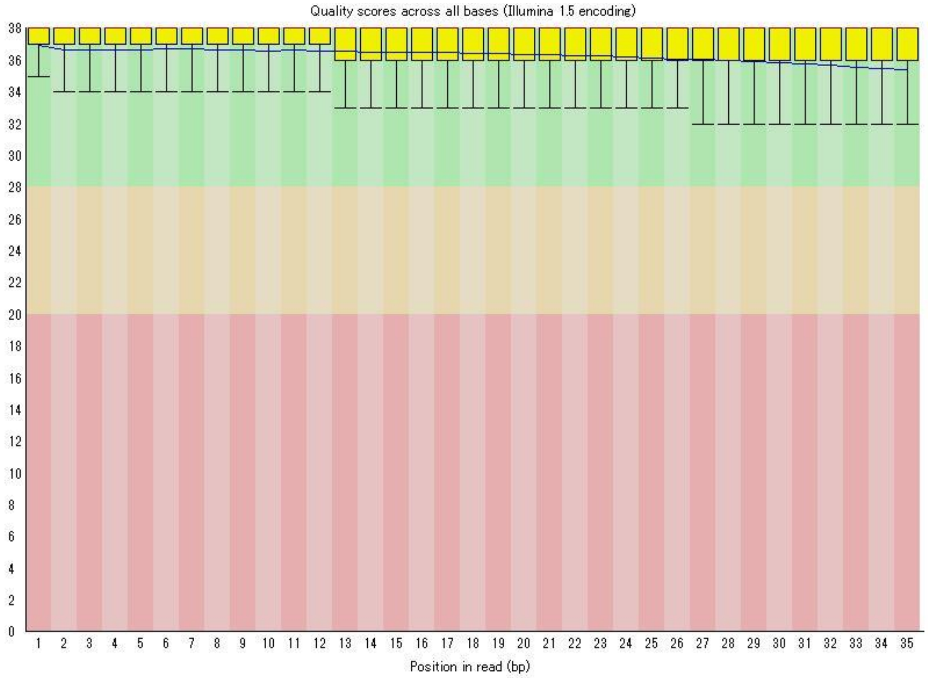

MyD88-Knockout mouse dendritic cell at t=24h after stimulation by LPS

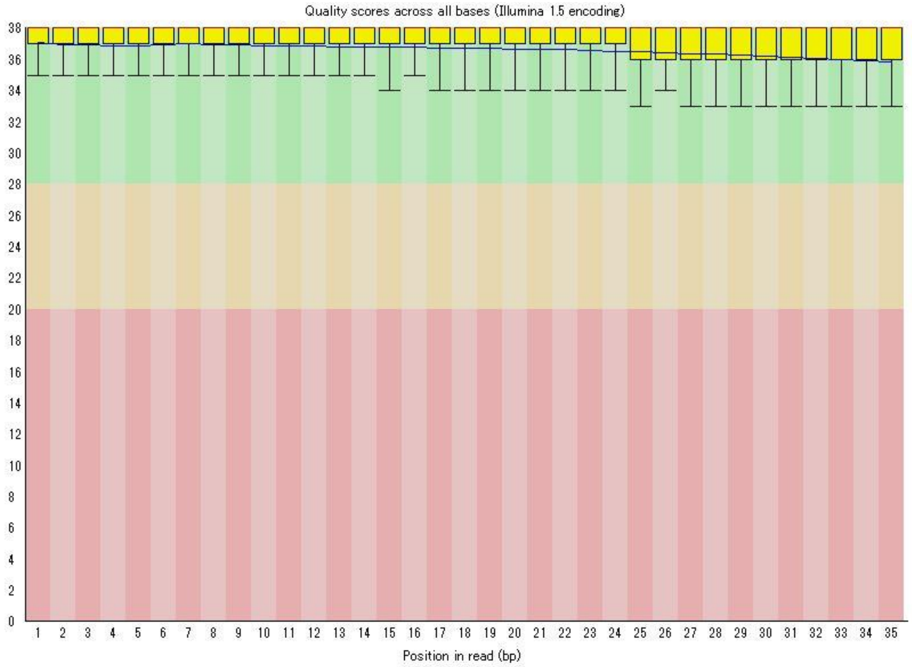

TRIF-Knockout mouse dendritic cell at t=0h before stimulation by LPS

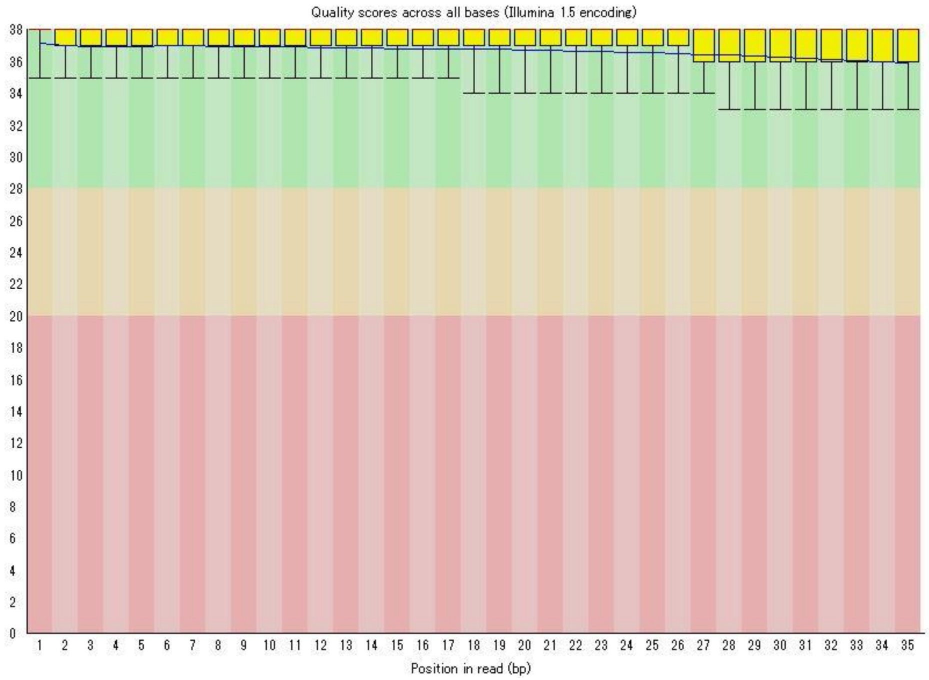

TRIF-Knockout mouse dendritic cell at t=0.5h after stimulation by LPS

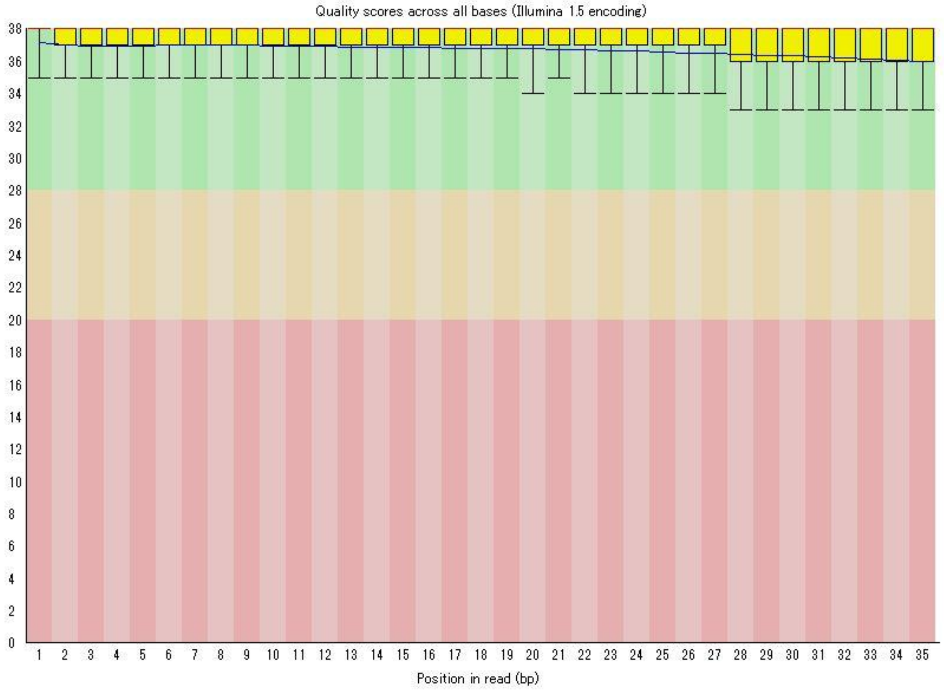

TRIF-Knockout mouse dendritic cell at t=1h after stimulation by LPS

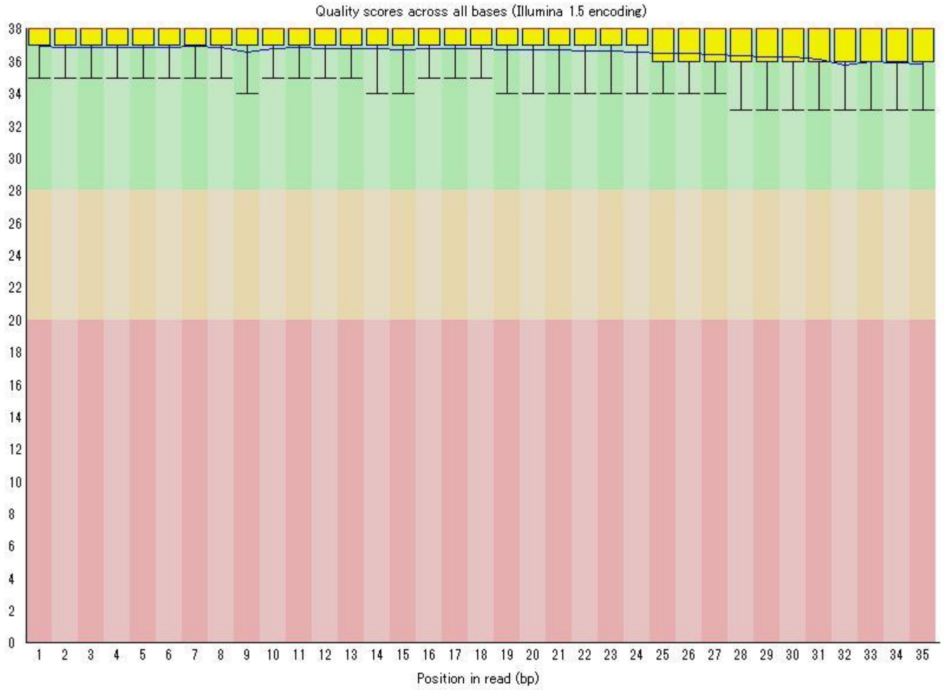

TRIF-Knockout mouse dendritic cell at t=2h after stimulation by LPS

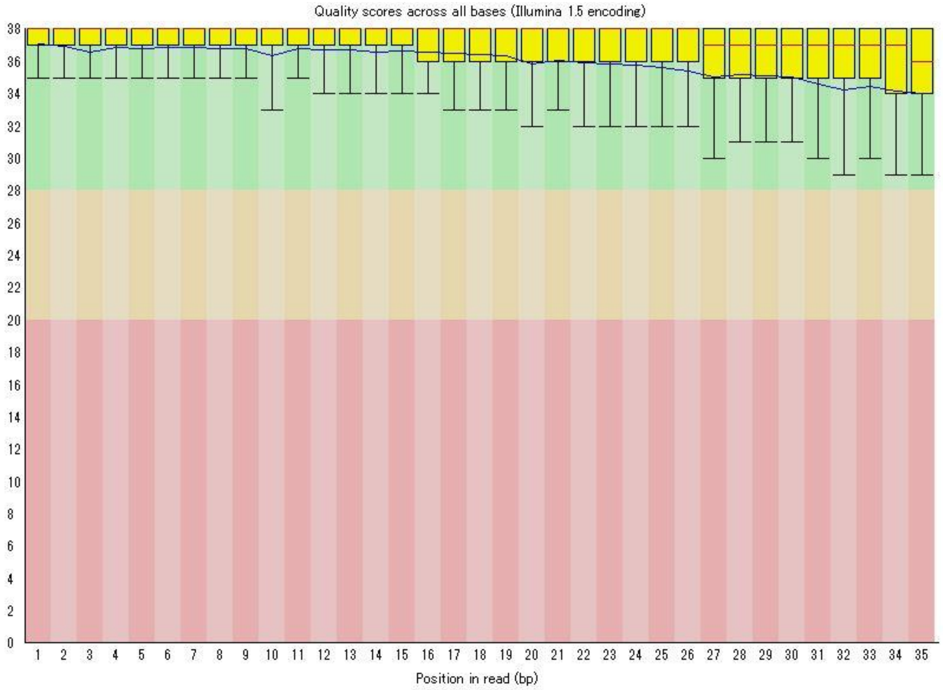

TRIF-Knockout mouse dendritic cell at t=3h after stimulation by LPS

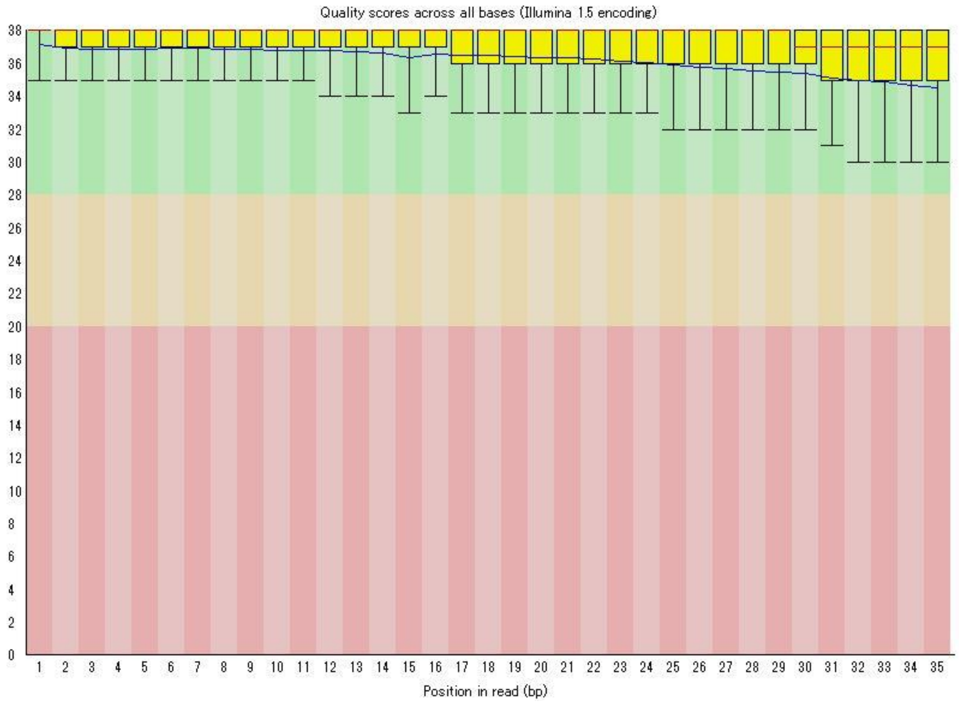

TRIF-Knockout mouse dendritic cell at t=4h after stimulation by LPS

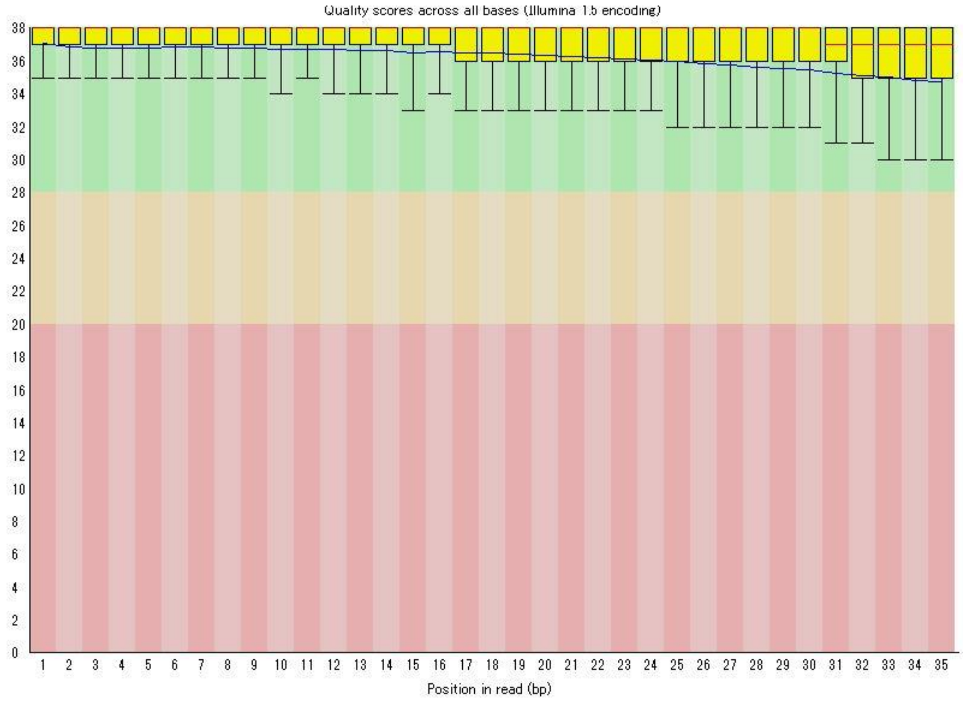

TRIF-Knockout mouse dendritic cell at t=6h after stimulation by LPS

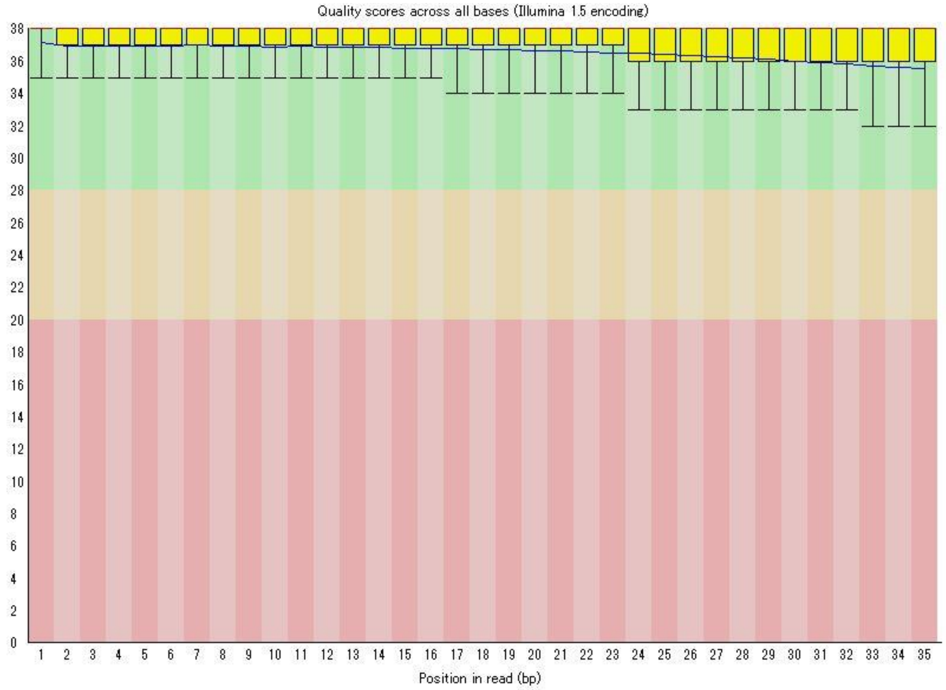

TRIF-Knockout mouse dendritic cell at t=8h after stimulation by LPS

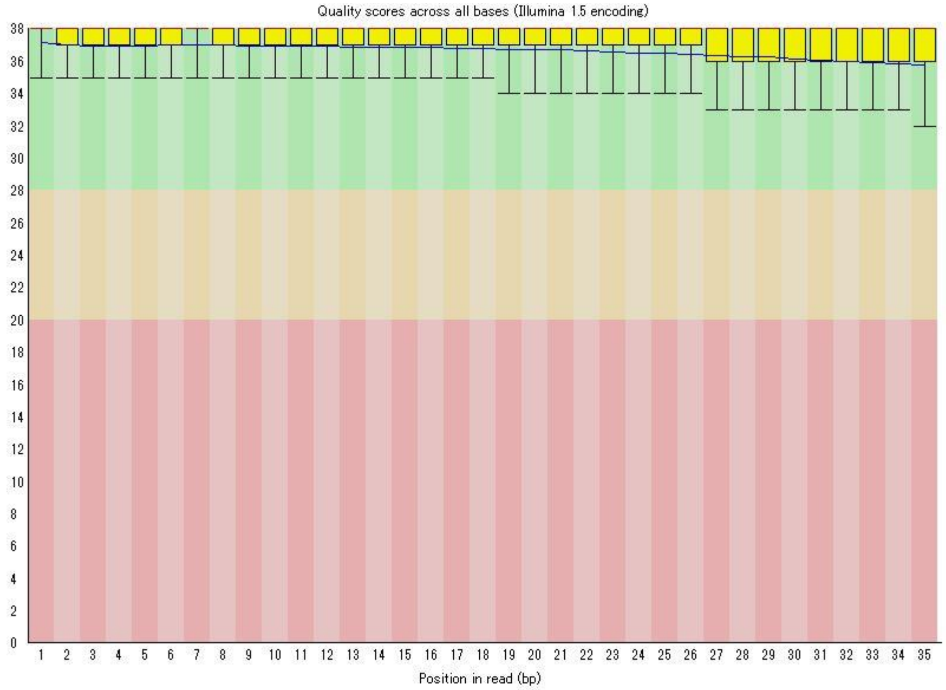

TRIF-Knockout mouse dendritic cell at t=16h after stimulation by LPS

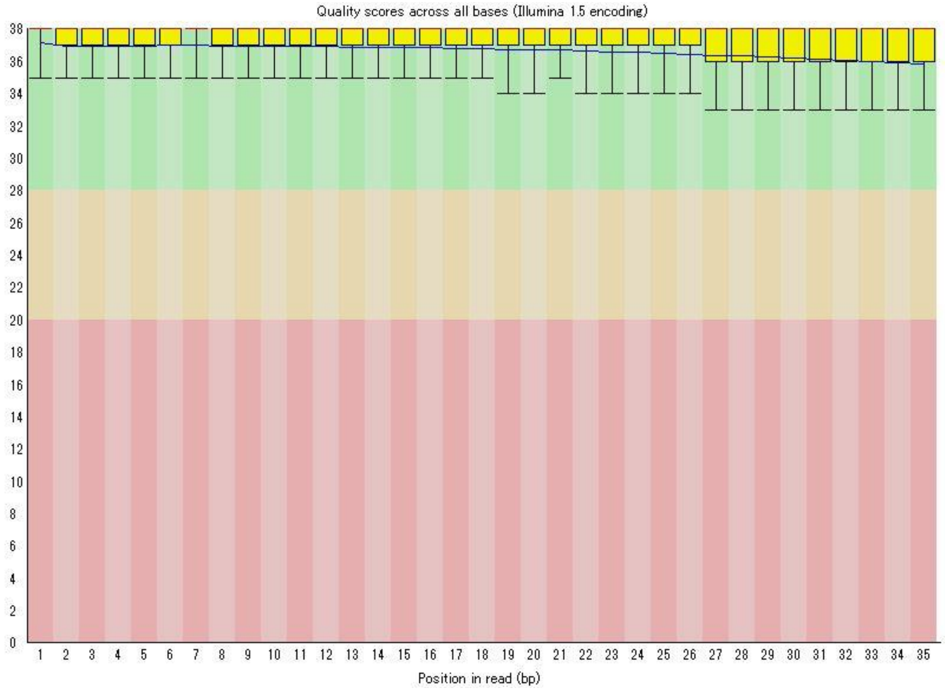

TRIF-Knockout mouse dendritic cell at t=24h after stimulation by LPS

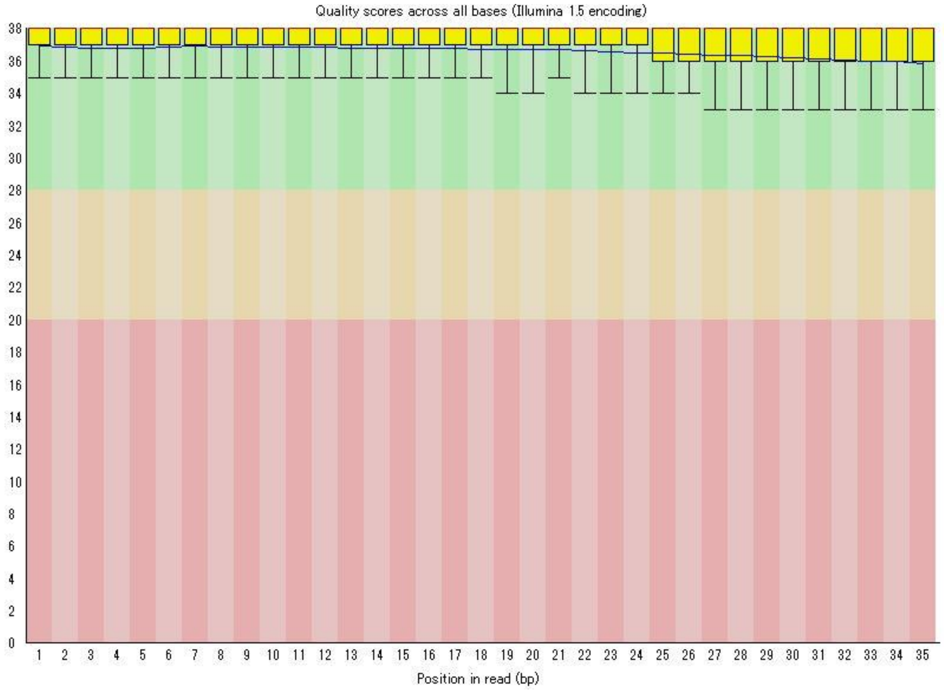

Mapping statistics for all RNA-Seq samples

	INPUT(# READS)	MAPPED(# READS)	PERCENT
**WT1**	29,574,369	28,591,092	96.7%
**WT2**	26,851,116	26,026,769	96.9%
**WT3**	29,590,750	28,727,192	97.1%
**WT4**	28,114,773	27,312,427	97.1%
**WT5**	27,743,993	26,983,977	97.3%
**WT6**	28,508,064	27,681,533	97.1%
**WT7**	29,125,057	28,386,723	97.5%
**WT8**	28,160,919	27,418,486	97.4%
**WT9**	27,696,542	26,858,879	97.0%
**WT10**	28,269,171	27,438,382	97.1%
**MYD1**	26,300,054	25,469,067	96.8%
**MYD2**	30,964,914	29,769,859	96.1%
**MYD3**	35,599,152	34,544,985	97.0%
**MYD4**	36,396,208	35,330,152	97.1%
**MYD5**	32,188,824	30,803,800	95.7%
**MYD6**	35,314,240	34,131,383	96.7%
**MYD7**	27,355,785	26,423,585	96.6%
**MYD8**	28,766,755	27,762,283	96.5%
**MYD9**	30,796,324	29,708,453	96.5%
**MYD10**	30,425,612	29,733,409	97.7%
**TRIF1**	30,103,165	29,260,806	97.2%
**TRIF2**	30,220,490	29,323,487	97.0%
**TRIF3**	29,724,933	28,877,578	97.1%
**TRIF4**	27,314,961	26,342,681	96.4%
**TRIF5**	28,540,742	27,780,497	97.3%
**TRIF6**	30,026,562	29,230,673	97.3%
**TRIF7**	29,189,831	28,481,689	97.6%
**TRIF8**	29,217,262	28,564,105	97.8%
**TRIF9**	29,803,100	29,111,180	97.7%
**TRIF10**	27,453,084	26,716,321	97.3%
